# Differentiation of adipose-derived stem cells to functional CD105^neg^ CD73^low^ melanocyte precursors guided by defined culture condition

**DOI:** 10.1186/s13287-019-1364-0

**Published:** 2019-08-09

**Authors:** Gabriela Zavala, Carolina Sandoval, Daniel Meza, Rafael Contreras, Walter Gubelin, Maroun Khoury

**Affiliations:** 1 Consorcio Regenero, La Plaza 2501, Las Condes, Santiago, Chile; 2 Laboratory of Nano-Regenerative Medicine, Faculty of Medicine, Universidad de los Andes, Monseñor Álvaro del Portillo 12455, Las Condes, Santiago, Chile; 3 Cells for Cells, La Plaza 2501, Las Condes, Santiago, Chile; 4 Biomedical Research Center (CIB), Faculty of Medicine, Universidad de los Andes, Monseñor Álvaro del Portillo 12455, Las Condes, Santiago, Chile; 5 Faculty of Medicine, Universidad de los Andes, Monseñor Álvaro del Portillo 12455, Las Condes, Santiago, Chile

**Keywords:** Melanocytes, ADSC, Vitiligo, Differentiation, MITF, Tyrosinase, Stem cells, CD105

## Abstract

**Background:**

The generation of functional human epidermal melanocytes (HEM) from stem cells provides an unprecedented source for cell-based therapy in vitiligo. Despite the important efforts exerted to obtain melanin-producing cells from stem cells, pre-clinical results still lack the safety and scalability characteristics essential for their translational application.

**Methods:**

Here, we report a rapid and efficient protocol based on defined culture conditions capable of differentiating adult adipose-derived stem cells (ADSC) to scalable amounts of proliferative melanocyte precursors (PreMel) within 30 days. PreMel were characterized in vitro through qPCR, Western blot, flow cytometry, biochemical assays, and in vivo assays in immunocompromised mice (NOD.Cg-Prkdc^scid^ Il2rg^tm1Wjl^/SzJ, or NSG).

**Results:**

After 30 days of differentiation, the stem cell-derived PreMel were defined as CD105^neg^ CD73^low^ according to immunophenotypic changes in comparison with parental stem cell markers. In addition, expression of microphthalmia-associated transcription factor (MITF), active tyrosinase (TYR), and the terminal differentiation-involved premelanosome protein (PMEL) were detected. Furthermore, PreMel had the potential to synthesize melanin and package it into melanosomes both in vitro and in vivo in NSG mice skin.

**Conclusions:**

This study proposes a rapid and scalable protocol for the generation of proliferative melanocyte precursors (PreMel) from ADSC. These PreMel display the essential functional characteristics of bona fide HEM, opening a new path for an autologous cellular therapy for vitiligo patients.

**Electronic supplementary material:**

The online version of this article (10.1186/s13287-019-1364-0) contains supplementary material, which is available to authorized users.

## Background

Human epidermal melanocytes (HEM) produce melanin, a pigment responsible for both skin color and protection from ultraviolet radiation (UVR) [1]. Melanin is synthetized in melanosomes and delivered to the surrounding keratinocytes, cells that reside in the epidermis and constitute the defense barrier against UVR [2].

HEM development and differentiation begin with the migration of multipotent progenitors from the neural crest. Expression of transcription factors including paired box gene 3 (PAX3), sex-determining region Y-box 10 (SOX10), lymphoid enhancer-binding factor 1 (LEF1), and microphthalmia-associated transcription factor (MITF) induce the commitment of progenitor cells into melanoblasts and subsequent differentiation into melanocytes when the cells reach the epidermis [3]. There is abundant literature regarding HEM human biology, which is fundamental for the understanding of processes relevant to their development and pathologies such as vitiligo.

Vitiligo is a pigmentation disease in which the malfunction or apoptosis of HEM generates the disappearance of melanin and, consequently, the generation of white macules in the skin and mucosa [4]. This damage to HEM is generally caused by autoimmune attack, redox unbalance, genetic predisposition, impaired cell adhesion, or dysfunction of the sympathetic nerves, but the consequences to the patient are similar no matter the cause [5]. Vitiligo cases can be classified into two main categories: segmental (SV) and non-segmental (NSV) vitiligo. SV includes cases with rapid stabilization in which the affected areas are not symmetric; these cases are in contrast to those of NSV, which are characterized by a high association with autoimmune disease, symmetric and bilateral macule distribution, and an uncertain disease course [6].

Skin grafts are currently the most promising treatment for stable vitiligo, via application of autologous HEM obtained from healthy zones of the skin directly after biopsy and isolation [7–10]. However, this technique faces one main limitation: the size of the skin biopsy limits the number of isolated epidermal cells and with it the efficiency of the therapy [11–13]. In an approach to transplant HEM expanded in cell culture [14–17], it has been found that HEM from vitiligo patients present low viability and difficulties for in vitro management [18–20]. This constraint drives the need to obtain large numbers of HEM or their progenitors without the invasive intervention of large, pigmented skin biopsies. Hence, stem cells represent an attractive source for cell transplantation therapies.

Research addressing this unmet medical need has thus far focused on the development of protocols to encourage HEM differentiation from stem cells. As a therapeutic strategy for vitiligo, the use of pluripotent stem cells including embryonic stem cells (ESC) [21–25], induced pluripotent stem cells (iPS) [26–28], and multilineage-differentiating stress-enduring (MUSE) cells [29, 30] has been explored. Both ESC and iPS displayed high differentiation efficiency but are still limited by safety [31, 32] and ethical issues (for ESC handling). In addition, economic limitations caused by high production costs make these options difficult to justify for a non-fatal disease. Further translational development of these techniques as viable treatment strategies for vitiligo patients has thus been obstructed.

Mesenchymal/stromal stem cells (MSC) have fueled ample research in the regenerative medicine field [33–35] due to their property to differentiate in vitro into adipocytes, osteoblasts, chondrocytes, and other cell types [36, 37]. MSC can be isolated from multiple post-natal and adult tissues, which opens the possibility of autologous therapies by allowing cell procurement from any patient. Safe use of MSC in humans has been demonstrated in several clinical trials [38, 39].

The differentiation of MSC into HEM has been addressed using cells from sources including dental pulp (DP-MSC) [40, 41] and bone marrow (BM-MSC) [42]. However, the differentiation protocols have been shown to be complex and lengthy (reprogramming times of 180–200 days), yielding a low number of HEM; hence, there is no effective protocol currently for the differentiation of adult MSC into HEM. Adipose-derived stem cells (ADSC) have been shown in studies to collaborate with HEM, promoting proliferation in vitro and grafting in vivo [43, 44].

In this work, we establish a new method for the efficient generation of HEM and their precursors from ADSC. The adipose tissue was selected as the source of choice due to its accessibility, differentiation potential, and clinical safety profile. ADSC differentiation will be guided by a defined culture condition developed by our laboratory in order to obtain a high number of melanocyte precursors or PreMel.

Furthermore, we hypothesize that the improvement of the engraftment can be achieved using proliferative HEM precursors rather than slowly dividing melanocytes. This can also insure a more effective repopulation and re-pigmentation of vitiligo spots.

## Methods

All the procedures presented in this work were approved by the Ethics Committee of Universidad de los Andes. All personnel involved in animal studies were trained internally using courses available at www.aalas.org, and assays were performed according to ARRIVE guidelines of the National Centre for the Replacement, Refinement & Reduction of Animals in Research (NC3Rs).

### Adipose tissue samples

Adipose tissue (AT) samples were obtained from elective cosmetic liposuction procedures and with the informed consent of donors. All AT donations were from healthy females (18–54 years old) with no reported vitiligo or other autoimmune diseases.

### ADSC isolation

ADSC were isolated and cultured as described in Zuk et al. [45]. Lipoaspirates were promptly processed the same day as liposuction under sterile conditions. The AT was transferred to several 50-mL conical tubes and was washed abundantly with phosphate-buffered saline (PBS) pH 7.4 in order to eliminate anesthetics and red blood cells. The AT was digested enzymatically using 0.1% collagenase type I (Thermo Fisher, MA, USA) for 60 min at 37 °C with gentle agitation. After several steps of centrifugation and washes, a cellular pellet (stromal vascular fraction, SVF) was obtained and was seeded in Dulbecco’s modified Eagle’s medium (DMEM, Corning, NY, USA) supplemented with 10% fetal bovine serum (FBS, Thermo Fisher), 2 mM l-glutamine, and 100 units/mL penicillin and 100 μg/mL streptomycin (both reagents from Thermo Fisher). The SVF from 50 mL lipoaspirate was seeded in one T175 cm^2^ flask. Non-adherent cells were discarded the next day, while the adherent ADSC were maintained in the previously described culture medium until 80% confluence, when they were harvested using 0.05% trypsin-EDTA (Thermo Fisher) and subcultured. ADSC were maintained in a humidified incubator at 37 °C and 5% CO_2_. All experiments were done using cells after four to six subcultures.

The human melanoma cell line A375 (ATCC® CRL-1619™) was kindly gifted from Dr. Manuel Varas (Universidad de los Andes). Fibroblasts were obtained after primary culture of dermal explants. Both cell types were maintained in the same culture conditions as ADSC.

### ADSC characterization

ADSC were characterized according to the International Society of Cellular Therapy (ISCT) and International Federation for Adipose Therapeutics and Science (IFATS) guidelines for MSC and ADSC, respectively [46, 47]. Expression of specific markers were evaluated by flow cytometry. Cells were harvested in 0.05% trypsin-EDTA and incubated in cytometry buffer containing specific antibodies (CD90-APC, CD105-PE, CD73-PB, CD44-FITC, CD34-APC, CD45-PECy^7^, HLA-DR-FITC, CD14-PE, CD31-PE, CD117-APC, CD146-FITC, CD13-APC, and CD106-PE) for 30 min at 4 °C. Samples were analyzed by BD FACS Canto II (BD Biosciences, CA, USA) and FlowJo software V10 (Tree Star, Ashland, OR, USA). Ten thousand events were collected for each sample, and doublets were discarded for analysis purposes.

The same antibody staining procedure was used to characterize PreMel and HEM. Changes between control and differentiated cell populations were determined by comparing the differences in the geometric mean fluorescence intensity (MFI) ratios for each surface marker, calculated as MFI ratio = MFI (specific marker)/MFI (autofluorescence).

For multipotent differentiation, ADSC or PreMel were seeded in 24-well plates at a density of 5 × 10^4^ cells/well and were stimulated with specific inductor media. For osteogenic induction, cells were treated with DMEM supplemented with 5% FBS, 2.17 mg/mL β-glycerophosphate (Sigma Aldrich, MO, USA), 50 μg/mL ascorbic acid (Sigma Aldrich), and 0.1 μM dexamethasone (Sigma Aldrich). Adipogenic induction was induced with DMEM supplemented with 5% FBS, 0.01 mg/mL insulin (Thermo Fisher), 100 μM indomethacin (Sigma Aldrich), 1 μM dexamethasone (Sigma Aldrich), and 0.25 mM isobutylmethylxanthine (Sigma Aldrich). In both protocols, cells were treated for 14 days. Chondrogenic differentiation was accomplished in 7 days with StemPro® Chondrogenesis Differentiation Kit (Thermo Fisher), according to the manufacturer’s guidelines.

Differentiation was determined using specific staining: Alizarin red S, Oil red O, and Safranin O dyes for osteogenic, adipogenic, and chondrogenic lineages, respectively.

ADSC were also characterized by fibroblast colony-forming unit frequency (CFU-F). ADSC were seeded between 0.5 and 50 cells/cm^2^ for 11 days. Later, the cells were fixed with 70% chilled methanol and stained with a 0.05% crystal violet (CV) solution. Colonies of more than 50 cells were counted under a magnifying glass, and CV was dissolved with 1% SDS to measure the absorbance at 540 nm using a spectrophotometer (Tecan Reader).

### HEM cell culture

HEM (C1025C, Thermo Fisher) were derived from neonatal foreskin and maintained in M254 medium (Thermo Fisher) supplemented with human melanocyte growth supplement-2 (HGMS-2) PMA-free (Thermo Fisher), which includes 0.2% bovine pituitary extract (BPE), 0.5% FBS, 3 ng/mL human recombinant FGF2, 0.5 μM hydrocortisone, 5 μg/mL insulin, 5 μg/mL transferrin, 3 μg/mL heparin, and 10 nM endothelin-1.

### ADSC differentiation into melanocytes lineage

ADSC were seeded at 2 × 10^3^ cells/cm^2^, and the next day, cell culture medium was eliminated and the cells were washed with PBS. Differentiation was induced with an induction medium developed in our lab composed of MCDB 153 (Sigma Aldrich) supplemented with 2% FBS, 2 mM l-glutamine, 100 units/mL penicillin and 100 μg/mL streptomycin, 0.5 μg/mL hydrocortisone (Sigma Aldrich), 50 μM DBcAMP (Sigma Aldrich), 1% insulin-transferrin-selenium (ITS-X) (Thermo Fisher), 4 ng/mL FGF2 (Thermo Fisher), and 20 ng/mL of αMSH (Sigma Aldrich). The growth medium was changed twice a week, and cells were subcultured at 80% confluence. Differentiation conditions were maintained for 30 days, after which cells were characterized.

### Quantitative real-time PCR analysis

Total mRNA was extracted from control and PreMel cells at different time points using TRIzol® Reagent (Thermo Fisher), and cDNA was synthesized by reverse transcription. The cDNA samples were analyzed by qPCR to determine the expression levels of different melanocyte markers using *GAPDH* as a normalizing gene (Table 1).

**Table 1:** Primers used for qRT-PCR

	Forward	Reverse
MC1R	TTGGACGCTTTCACGCTCTG	TCTGGAAACTGAGTGAGCCCT
MITF-M	CAGCGTGTATTTTTCCCACAG	ATGTCTGGATCATTTGACTTGGG
MITF-T	ACTTTAGCTTTCTGAGCAAGAGG	CCAAGGAAGTCACAGGCATAAA
PAX3	TCCGAGACAAATTACTCAAGGAC	TCACCTTTCCCGAATTTACTTCTC
PAX6	CCAGCAACACACCTAGTCATATTC	GCCAGATGTGAAGGAGGAAAC
PMEL	AACTCCAGAGGCTACAGGTATG	GTCTCCACCCACTCTGTAGTT
SOX2	GGAGAGAGAAAGAAAGGGAGAGA	GCCGCCGATGATTGTTATTATTAT
SOX10	ACTTTAGCTTTCTGAGCAAGAGG	CCAAGGAAGTCACAGGCATAAA
TRP2	GTACGACAGAGACAAGGAAAGTAAG	CCCAAGCAACTGAGCAGAAA
TYR	GATTGTCTGTAGCCGATTGGAG	GGGTTCTGGATTTGTCATGGT
IL-6	AAATTCGGTACATCCTCGACGGCA	AGTGCCTCTTTGCTGCTTTCACAC
TGF-β1	TGGACTTCGGCCACATCAAGAAGA	TGTTGTAAAGGGCCAGGACCTGAT
GAPDH	CAGCCTCAAGATCATCAGCA	CATGAGTCCTTCCACGATAC

### Immunofluorescence detection of MITF, PMEL, and TYR

After 30 days of differentiation, PreMel were seeded in glass coverslips and after 24 h were fixed in 4% paraformaldehyde (PFA) for 15 min at room temperature. The cells were permeabilized and blocked in PBS containing 0.2% Triton X-100 and 1% bovine serum albumin (BSA) for 30 min. The cells were incubated with respective antibodies: mouse anti-human PMEL (HMB45 clone, #cat M0634, DAKO, RRID:AB_2335682), rabbit anti-MITF (cat# 122982, Abcam, RRID:AB_10902226), or mouse anti-tyrosinase (#cat sc20035, Santa Cruz, RRID:AB_628420) at 4 °C overnight. After the incubation, the cells were washed in PBS and stained with the secondary antibodies: goat anti-mouse DyLight® 488 (35502, Thermo Fisher) or goat anti-rabbit DyLight® 594 (35561, Thermo Fisher). 4′, 6-Diamidino-2-phenylindole (DAPI, Sigma Aldrich) was used for nuclear staining. Coverslips were mounted in FluorSave™ (Merck Millipore, MO, USA) prior to fluorescence microscopy analysis (ECLIPSE TE2000U, Nikon).

### Western blot for MITF and TYR determination

Cell lysates were obtained from control and differentiated cells. At different time points, the cells were washed with cold PBS, lysis buffer was added, and a cell scraper was used for optimized lysis. The lysis buffer contained 70 mM SDS, 50 mM Tris-HCl pH 6.8, and 10% glycerol and was enriched with Protease Inhibitor Cocktail (Sigma Aldrich). Lysates were centrifuged for 10 min at 10,000×*g* and 4 °C. A Bradford assay (BioRad, CA, USA) was used for protein quantification. SDS-polyacrylamide gel electrophoresis of cell lysates was conducted using a 7.5% acrylamide gel. Thirty to 50 μg cell lysate was loaded into each well with 5 μL protein standard (Precision Plus Protein™ Kaleidoscope™ Prestained Protein Standards, BioRad). Proteins were subsequently transferred to a 0.45-μm pore nitrocellulose membrane.

The membranes were blocked with 5% BSA in 0.1% Tris-buffered saline buffer-Tween 20 (TBST) for 1 h at room temperature. The membranes were incubated overnight at 4 °C in blocking solution with the primary antibodies anti-MITF (1/3000), TYR (1/500), or rabbit anti-vinculin (1/5000, #cat 129002, Abcam, RRID:AB_11144129). After several washes, the membranes were incubated with the secondary antibody goat anti-mouse IgG Alexa Fluor® plus 680 (A32729, 1/15000, Invitrogen), or goat anti-rabbit IgG Alexa Fluor® plus 800 (A32735, 1/25000, Invitrogen) for 1 h at room temperature in the dark. Finally, the membranes were washed four times in PBS and revealed using the Odyssey® CLx Imaging System. For quantification and image analysis, Image Studio™ Lite software was used.

### Proliferation assay

1 × 10^3^ cells were seeded in a 96-well plate in a specific medium (DMEM 10% FBS for ADSC, inductive medium for PreMel, and M254 supplemented with HMGS-2 for HEM). Cell proliferation was estimated through a WST-1 assay (Quick Cell Proliferation Assay Kit, BioVision, CA, USA) according to the manufacturer’s guidelines. Briefly, the cells were maintained in 100 μL of their specific medium, and 10 μL of WST-1 was added per well. The cells were incubated at 37 °C in a cell incubator for 2 h, and the supernatant was transferred into a 96-well flat-bottom plate to measure the absorbance at 450 nm (620 nm reference) in a spectrophotometer (Tecan Reader). The seeded cell culture plate was washed one time with PBS, and fresh medium was added to continue the cell culture. UVR (type B radiation) treatment was done 24 h after cell seeding: cell medium was discarded, and each well was washed with PBS, then 50 μL PBS was added per well. The 96-well plate was placed under a UVB lamp (UVM57 lamp, UVP Ultra-Violet Products, USA) for 10 or 20 s of illumination (each second is equivalent to approximately 1 mJ/cm^2^). PBS was then discarded and replaced with fresh medium to continue the cell culture until the end of the experiment. Oxidative stress was induced with H_2_O_2_ (Merck Millipore) at different concentrations in the cell-specific media, and the cells were stimulated at the fourth day after seeding with 0, 50, 100, 300, and 500 μM H_2_O_2_ for 24 h. After the stimulus, the medium was eliminated, cells were washed, and fresh medium was added. The supernatants were recovered for spectrophotometer measurements. For each experiment, the cells were maintained in cell culture conditions until the end of the assay. Population doubling time (PDT) and growth rate (GR) were determined with the Exponential Growth Curve tool from GraphPad Prism version 5.0b for Windows (La Jolla, CA, USA).

### L-DOPA (3, 4- dihydroxyphenylalanine) assay

TYR enzymatic activity was determined through the L-DOPA assay. Qualitative cell staining and quantitative L-DOPA oxidation measurements were performed. Cells were seeded at 5 × 10^3^ cells/cm^2^ for L-DOPA assay staining; next day, the medium was discarded, and the cells were fixed in 4% PFA. Cells were then incubated with fresh 0.1% L-DOPA in PBS (pH 6.8) at 37 °C for 2 h. The samples were washed in PBS and photographed with the Olympus CX41 microscope. For L-DOPA determination in cell lysates, cells were washed with PBS and lysed with a minimum volume of lysis buffer (1% Triton X-100 in PBS pH 6.8). Cells were frozen at − 80 °C for at least 20 min and were thawed for 10 min at room temperature in gentle agitation, lysed mechanically with a pipette tip, and centrifuged at 13,000×*g* for 15 min at 4 °C. The supernatants were collected, and absorbance was measured at 595 nm for protein quantification via the Bradford method. 0.1% L-DOPA was prepared fresh in PBS pH 6.8, and 10 μL of this substrate was added to 25 μg proteins suspended in 90 μL total volume. Samples were incubated at 37 °C, and absorbance was measured at 475 nm each 30 min for 10 h. All measurements were made in a NanoQuant Infinite TECAN M200 PRO.

### Melanin determination

For cellular melanin determination [48], cells were washed in PBS, trypsinized, and counted. The cells were lysed in 1 M NaOH with 10% DMSO at 80 °C for 1 h. After centrifugation at 10,000×*g* for 5 min, absorbance was measured at 415 nm using a standard curve of synthetic melanin (Sigma Aldrich). All measurements were normalized according to cell number or protein concentration.

### Transmission electron microscopy

Localization and stage of melanosome development were determined by TEM as described by Paino et al. [40]. Cells were fixed in 2.5% glutaraldehyde in 0.1 M cacodylate buffer and post-fixed in 1% OsO_4_. After dehydration, the samples were mounted for analysis with SEM LEO 1420VP transmission electron microscope (Laboratorio de Microscopía Electrónica de Barrido SEM, Pontificia Universidad Católica de Chile).

### PreMel transplantation

To evaluate PreMel grafting and melanin synthesis in vivo, cells were injected into the dorsal skin of 8-week-old immunocompromised male mice (NOD.Cg-Prkdc^scid^ Il2rg^tm1Wjl^/SzJ or NSG) according to the methods described by Alexeev and Yoon [49]. Mice were anesthetized with 3–5% sevoflurane inhalation, and hair removal was performed using an electric shaver and a hair removal cream for 2 min. 1 × 10^6^ cells (PreMel, HEM or ADSC) were suspended in 50 μL saline and were applied to the mice intradermally using a tuberculin syringe, with three injections per animal. Animals were maintained under standard conditions (water and food ad libitum) and after 2 weeks were euthanized with CO_2_ inhalation for skin analysis.

### Melanin determination in mouse skin

Melanin determination was made according to the methods of Yamamoto et al. [50] with modifications. After euthanasia, the portion of the skin that was injected with cells was extracted, weighed, and boiled in 10 mL 0.1 M acetic acid for 10 min. When the suspension was cooled, absorbance was measured at 415 nm. The following steps from the original protocol were not completed due to the vascularization of the back of the skin, which could have generated excessive noise in the absorbance measurements following complete digestion of the tissue, especially when the expected results were discrete.

### Immunohistochemistry of PreMel grafted in mouse skin

After the in vivo assay, the mouse skin was removed and fixed in 10% formalin solution (Sigma Aldrich) for 16 h at room temperature. After several washes with PBS, the tissue was dehydrated using a battery of ascending concentrations of alcohols and xylene, after which tissues were embedded in paraffin. Samples were cut into 5-μm sections in a microtome. For immunohistochemistry, slides were stained with the antibody anti TYR (permeabilization needed) or CD90-FITC. DAPI was used for nuclear staining, and samples were mounted with Fluorsave. Subsequently, slides were analyzed with the Sp8 confocal microscope (Leica) and LAS X software (V 3.3.0.16799).

### Fontana-Masson staining

Melanin determination in mouse skin after PreMel grafting was done using the Fontana-Masson staining kit according to the manufacturer’s instructions (Abcam). Briefly, samples were incubated with 10% pre-warmed silver nitrate solution at 60 °C for 60 min. After washes and posterior treatment, the samples were counter-stained with Nuclear Fast Red for nuclear visualization. Pictures were taken with the Olympus CX41 microscope.

### Statistics

All assays were performed at least in triplicate using three to five different human donor samples. Values are shown as mean ± SEM, and statistical significance was estimated using Student’s unpaired *t* test or analysis of variance (ANOVA), *P* < 0.05 was considered as statistically significant. The software GraphPad Prism 5.0b was used for statistical analysis.

## Results

### PreMel display a combined immunophenotype of ADSC and HEM surface markers

ADSC are isolated from the heterogeneous SVF of adipose tissue and need to be characterized according to the guidelines from the ISCT and IFATS to be considered MSC [46, 47]. First, we analyzed the expression of several classic mesenchymal markers. ADSC were positive for CD105, CD90, CD73, and CD13 in more than 95% of the cell population; CD44 was also positive in a minority of the population (Additional file 1: Figure S1A-E). Likewise, ADSC are negative for CD31, CD14, CD45, and CD106 and partially positive for CD34 (Additional file 1: Figure S1F-J). Additionally, ADSC have the potential to differentiate into adipogenic, chondrogenic, and osteogenic tissue in vitro in response to specific inductive media as was shown by positive lineage-specific stainings: Red Oil O, Safranin O, and Alizarin Red S, respectively (Additional file 1: Figure S1K-M). The capacity for ADSC self-renewal was estimated using a CFU-F assay showing the progenitor frequency (Additional file 1: Figure S1N). All these properties taken together confirm the MSC signature of ADSC.

As ADSC present a differentiation potential in vitro, we developed a defined medium to induce the differentiation of ADSC into HEM and evaluated the changes in the expression of the abovementioned markers by flow cytometry (details of the gating strategy are displayed in Additional file 2: Figure S2).

**Fig. 1:**
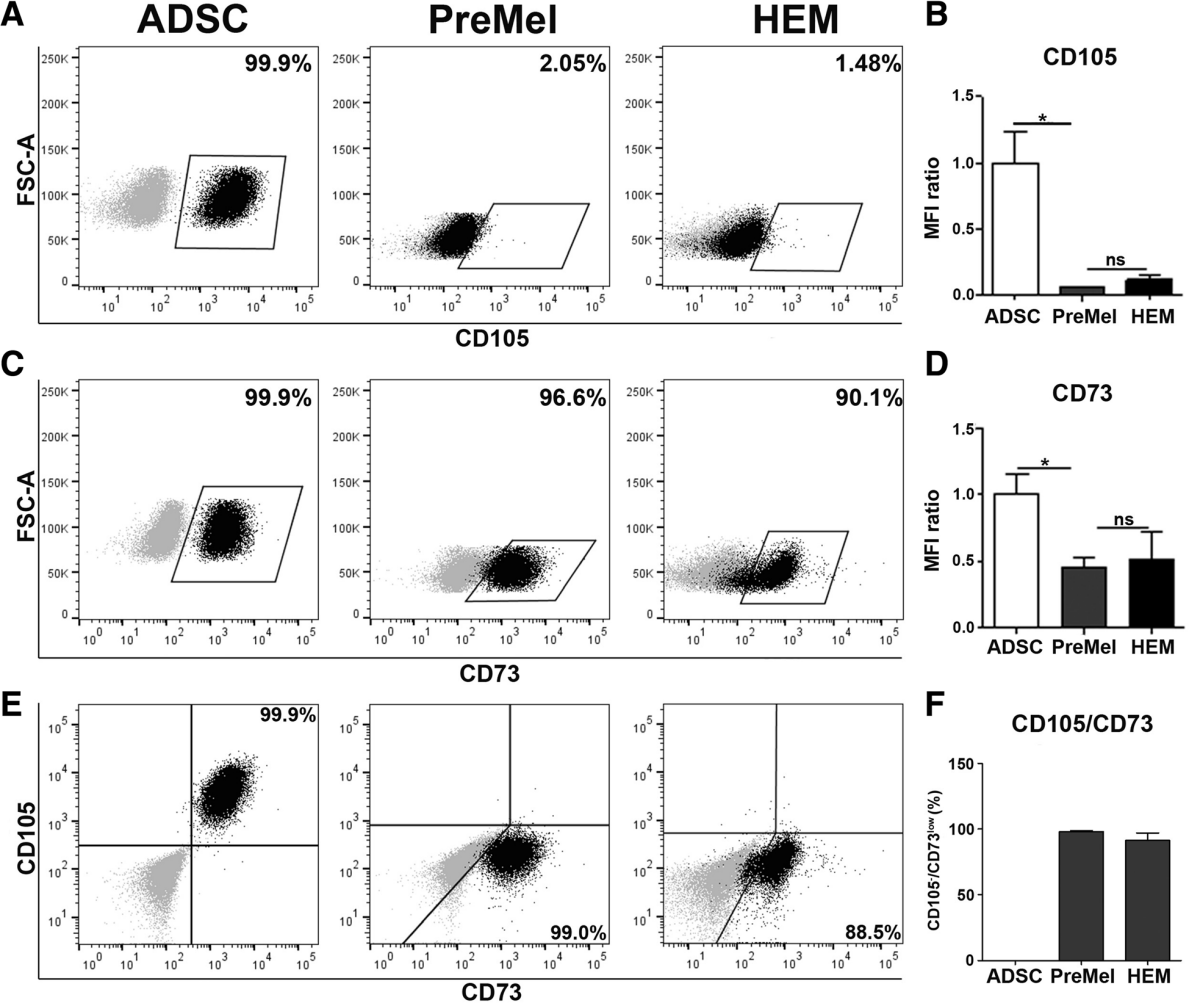
Evaluation of MSC classical markers at 30 days post-differentiation. After the induction protocol, the expression of MSC markers were evaluated by FACS. **a** CD105 is lost in PreMel after differentiation and reaches similar levels to HEM, dot plot shows the percentage of positive cells. **b** MFI ratio of CD105 in ADSC, PreMel, and HEM. **c** Dot plots shows the high percentage of CD73-positive cells in ADSC, PreMel, and HEM. **d** MFI ratio shows that CD73 expression decreases by 50% in PreMel compared to ADSC. **e** Distribution of CD105 and CD73 expression in ADSC, PreMel, and HEM. **f** Percentage of CD105^−^ CD73^low^ cells in ADSC, PreMel, and HEM. MFI ratio was calculated as MFI (specific staining)/MFI (autofluorescence) for each marker. Data from PreMel and HEM were normalized with respect to undifferentiated ADSC. For dot plots, gray dots represent autofluorescence control, and black dots represent stained cells with their respective marker. *n* = 3, *P < 0.05 one-way ANOVA

CD105 was highly expressed in ADSC (99.8% ± 0.12% positive cells), but it was absent in PreMel (0.36% ± 0.25% positive cells). Notably, HEM also expressed very low and variable levels (11.9% ± 7.83%) of CD105 (Fig. 1a, Table 2). The MFI ratio also showed that the level of expression of CD105 diminished significantly in PreMel with respect to ADSC and was very similar to HEM (Fig. 1b).

**Table 2:** Expression of CD105 and CD73 in ADSC, PreMel, and HEM (percentage of positive cells ± SD)

	CD105^+^ CD73^+^	CD105^+^	CD73^+/low^	CD105^neg^ CD73^low^	CD105^neg^ CD73^neg^
ADSC	99.2 ± 0.53	99.8 ± 0.12	99.4 ± 0.52	0.11 ± 0.20	0.12 ± 0.2
PreMel	0.29 ± 0.21	0.36 ± 0.25	98.2 ± 1.46	98.2 ± 1.46	1.78 ± 1.44
HEM	11.4 ± 8.23	11.9 ± 7.83	91.4 ± 9.87	91.4 ± 9.87	7.65 ± 9.67

Furthermore, a 50% decrease in the MFI ratio was also observed for the expression of CD73 in PreMel with respect to ADSC, albeit the entire population remained positive (98.2% ± 1.46%) (Fig. 1c, Table 2). Both MFI ratio and percentages were comparable with HEM expression, reaching a similar level (91.4% ± 9.87%) (Fig. 1c, d).

Other markers, such as CD44 and CD146, that were positive in ADSC remained unchanged following the differentiation protocol (Additional file 3: Figure S3A-B, 3E-F, respectively). A slight decrease was detected for CD90 expression in PreMel, but it was not statistically significant (Additional file 3: Figure S3C-D). CD117 expression increased in PreMel, but the percentage of positive cells was too low to be considered significant (Additional file 3: Figure S3G-H). CD45, CD31, CD14, and HLA-DR were not detected in ADSC, PreMel, or HEM (data not shown).

An important change of CD105 and CD73 expression was detected following the differentiation protocol (Fig. 1e, f; Table 2). The co-expression of these two markers in PreMel was notably similar to that in HEM (98.2% ± 1.46% vs 91.4% ± 9.87%, respectively) but differs significantly from that in ADSC (0.11% ± 0.20%). Hence, PreMel cells were designated as the CD105^neg^ CD73^low^ cell population.

Since adipogenic differentiation of ADSC is another gold standard property of MSC (Additional file 1: Figure S1M), we evaluated whether PreMel retained this potential following the 4-week induction protocol. Interestingly, PreMel displayed a significantly lower number of Oil Red O^+^ cells in comparison with ADSC (Additional file 4: Figure S4). The decrease in the adipogenic differentiation potential of PreMel along with the diminished expression of CD105-CD73 with respect to ADSC are indicators of the loss of their MSC identity and suggest a change of their cell fate.

### Rapid and efficient differentiation of ADSC into PreMel

**Fig. 2:**
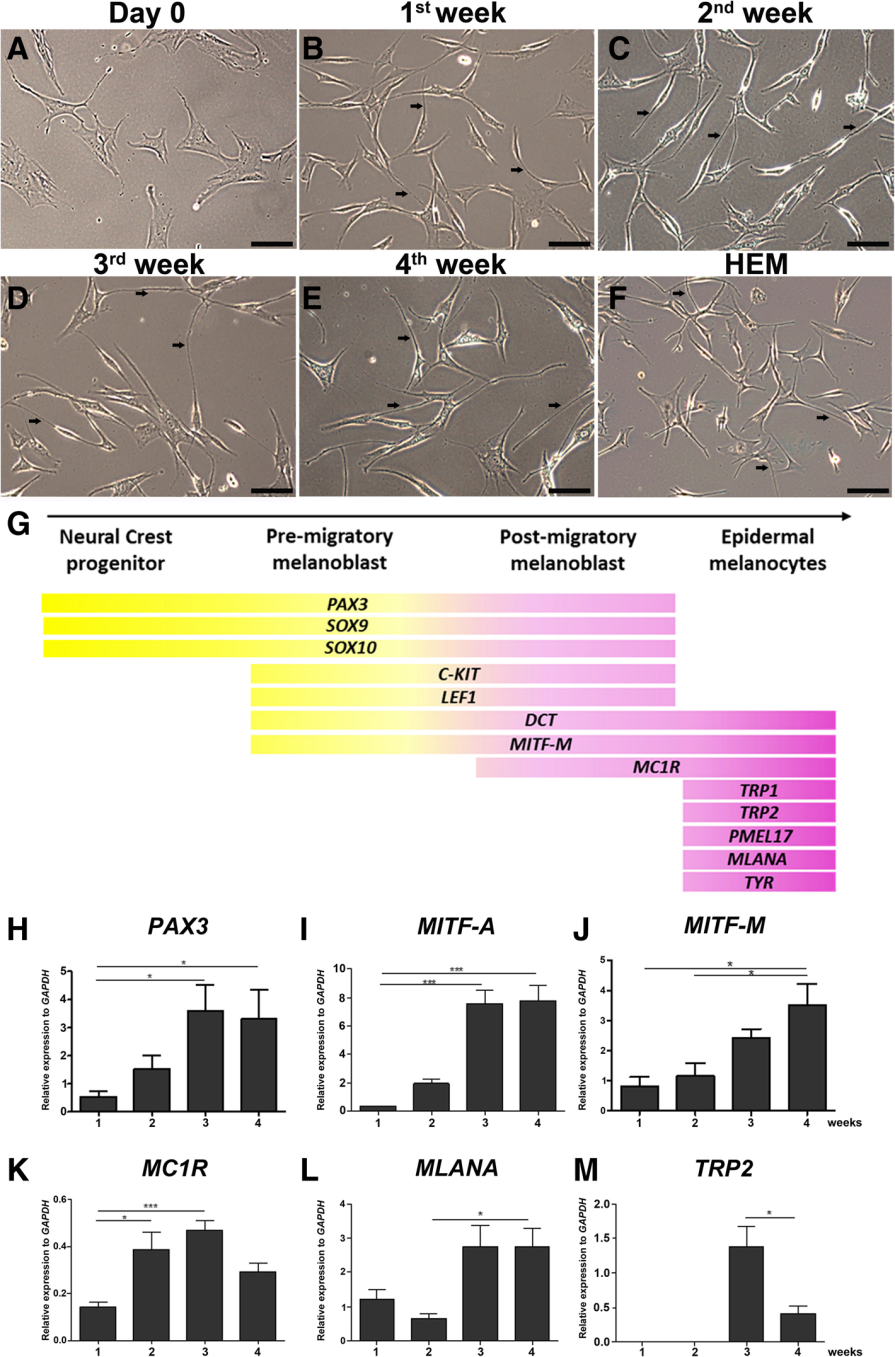
PreMel and HEM share a common gene transcription pattern. **a** Phase-contrast images show ADSC fibroblastoid morphology in control conditions. **b** After 1 week of differentiation, long and thin cellular extensions are observed. **c** Along with these cellular protrusions, the entire cells look thinner with respect to **a**. This phenotype continues after the third (**d**) and fourth (**e**) week and resembles the phenotype of HEM (**f**). Black arrows highlight the long cellular protrusions. Scale bar = 250 μm. **g** Scheme of different stages and gene expression of HEM during mammalian differentiation. Some of these genes were evaluated during ADSC differentiation protocol. **h** *PAX3* shows a constant increment during the protocol with a significant increase after 3 and 4 weeks. The same pattern is seen with **i** *MITF-A* and **j** *MITF-M*. **k** *MC1R* transcript increases significantly from the second week. **l** *MLANA* appears mostly during the second part of the protocol. **m** *TRP2* expression is detected after 3 weeks of differentiation. *n* = 3, *P < 0.05 one-way ANOVA; statistical analysis was calculated with respect to the first week of differentiation. Non-differentiated ADSC were used as a control

We then evaluated whether the loss of the MSC signature in PreMel was compensated with a gain in HEM features. ADSC were stimulated for 4 weeks, and cell morphology and gene expression were evaluated. The shape of ADSC in cell culture was generally fibroblastoid (Fig. 2a). After the first week, a change in cell morphology was observed where the fibroblastoid shape became more elongated and displayed bipolar forms (Fig. 2b). After 2 weeks, the cells acquired a bipolar shape with long processes (Fig. 2c). The observed phenotype was even pronounced after 3–4 weeks (Fig. 2d, e) ,and cell morphology was highly heterogeneous between ADSC-like cells and melanocyte-like cells (Fig. 2f).

### Molecular signature of PreMel

During the differentiation of HEM, each stage is characterized by the expression of specific genes (Fig. 2g). To evaluate the differentiation of ADSC into PreMel, we analyzed a panel of genes during the induction protocol: *PAX3*, *MITF-A*, *MITF-M*, melanocortin receptor 1 (*MC1R*), protein melan-A (*MLANA*), and tyrosinase-related protein 2 (*TRP2*). Other genes were also evaluated, but transcripts barely reached detection level in PreMel (data not shown).

The differentiation protocol induced a continuous increase of *PAX3*, *MITF-A*, and *MITF-M* reaching a maximum during the third to fourth week (Fig. 2h–j). Additionally, we analyzed the levels of *MC1R*, *MLANA*, and *TRP2* which represent later stages of HEM development. *MC1R* increased since the beginning of the differentiation protocol, reaching a maximum in the third week (Fig. 2k). Also, there was an increment for both *MLANA* and *TRP2* since the third week (Fig. 2l, m).

In addition, a more complete panel of genes was evaluated in HEM and A375, an amelanotic melanoma cell line, as positives control (Additional file 5: Figure S5).

### PreMel express the melanocyte-specific proteins: MITF, TYR, and PMEL

To determine the commitment level of the PreMel population within the melanocyte lineage stages, we analyzed the expression of melanocyte-specific proteins: MITF as a marker for the entire lineage (progenitors and HEM), and TYR and PMEL for terminal differentiation.

While MITF expression was detected in the entire cell population of PreMel and HEM by immunofluorescence and FACS (Additional file 6: Figure S6), we were unable to determine whether the protein corresponded to the isoform A or M; hence, we analyzed the MITF expression by Western blot.

**Fig. 3:**
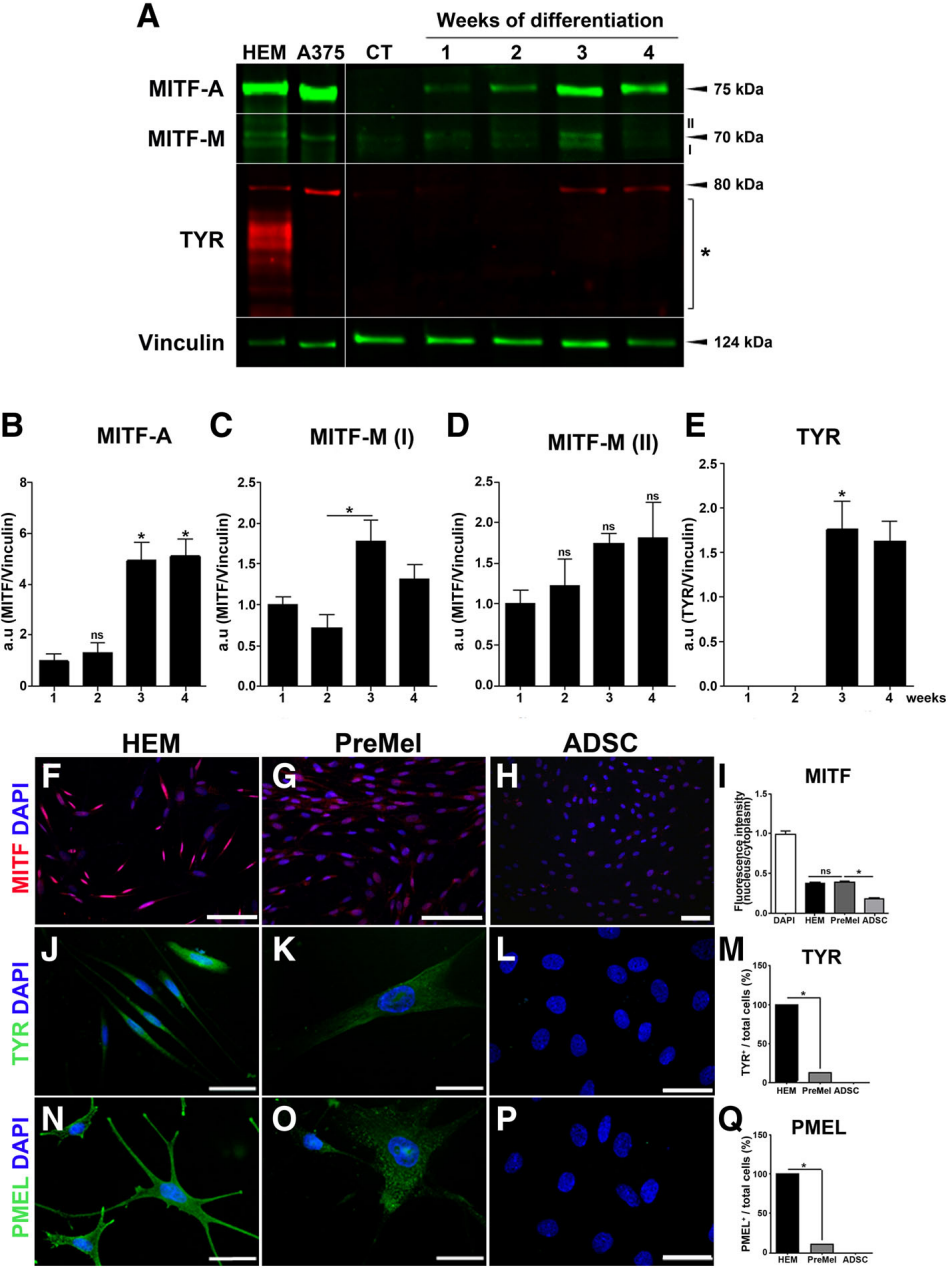
PreMel express HEM-specific proteins. Expression of MITF and TYR was evaluated by Western blot in HEM, A375 cells, ADSC, and PreMel. **a** In HEM and A375 cells, there is a band at 75 kDa and a doublet at 70 kDa, which represent MITF-A and MITF-M isoforms, respectively. Bands were denominated as I (lower band) or II (upper band) only for quantification. The reported band for *N*-glycosylated TYR is 80 kDa, but in HEM, multiple bands (*) appear that correspond to less *N*-glycosylated forms of the enzyme. Vinculin was used as a loading control. **b** Quantification shows that there is a significant increase in MITF-A levels in PreMel. **c**–**d** Each band of the MITF-M doublet was quantified as I and II. **e** TYR protein levels rise in response to the differentiation induction. **f** MITF localization in HEM is both nuclear and cytoplasmic. **g** MITF distributes similarly in PreMel compare to HEM. **h** ADSC express lower levels of MITF. **i** Nucleus/cytoplasm ratio of fluorescence intensity of MITF shows that the cellular distribution of MITF is similar between PreMel and HEM. Data are normalized with respect to DAPI intensity. TYR localization is cytoplasmic in **j** HEM and **k** PreMel, but not all differentiated cells are positive for TYR. **l** In ADSC, TYR is not detectable. **m** Quantification of TYR^+^ HEM, ADSC, and PreMel. A similar pattern is observed for PMEL in **n** HEM and **o** PreMel, with a low percentage of positive cells. **p** There is no expression of PMEL in ADSC. **q** Quantification of PMEL^+^ HEM, ADSC and PreMel. Scale bar = 100 μm (**f**–**g**) and 50 μm (**j**–**p**). *n* = 3, *P < 0.05 one-way ANOVA, compared to ADSC

In HEM lysates, we detected two isoforms of MITF according to their molecular weight (MW): one band at 75 kDa and two bands with a MW close to 70 kDa. The 75-kDa band corresponds to the MITF-A isoform, and the doublet corresponds to MITF-M (specifically expressed in HEM) in different phosphorylation states (Fig. 3a). In the cell line A375, both isoforms were detected. MITF-A and MITF-M were almost absent in ADSC but appeared during the first week of induction in PreMel, with a constant increase until the fourth week as shown in the quantification plot (Fig. 3a–c). These results evidence the inductive effect of the differentiation protocol through the activation of the signaling pathways that command the transcription and translation of the MITF gene, and we were able to detect both A and M isoforms in PreMel.

Immunofluorescence analysis showed the distribution of MITF; in HEM and PreMel, there was a strong nuclear stain, but the protein was also visualized in the cytoplasm. In ADSC, we observed the protein both in the cytoplasm and the nucleus but in a minority of cells (Fig. 3f–h).

Analyzing the expression of MITF by immunofluorescence and Western blot results altogether pointed at the expression of MITF-A and MITF-M by both HEM and PreMel (Fig. 3a, f, g). However, the cellular distribution of the specific isoforms was not determined, which can be relevant to suggest the transcriptional activity of MITF-M in PreMel. For this, we measured the fluorescence intensity of MITF in the cytoplasm and nuclei of HEM, ADSC, and PreMel in order to determine if the distribution of the transcription factor in PreMel was similar to HEM or ADSC. DAPI was used as an absolute nuclear staining control. In HEM, MITF was distributed both in the nucleus and the cytoplasm, with higher enrichment in the latter. A similar distribution was detected in PreMel, and a more pronounced cytoplasmic distribution was detected in ADSC (Fig. 3i). This result showed that the differentiation process induced an accumulation and redistribution of MITF in the nuclei of PreMel similar to its distribution in HEM, as both isoforms were expressed in PreMel (according to the Western blot). Therefore, MITF-A can be accumulated as much as MITF-M in the nucleus in an unknown proportion.

TYR and PMEL are HEM-specific proteins and transcriptional targets of MITF-M [51, 52]. Therefore, we analyzed their expression in ADSC, PreMel, and HEM cells. TYR expression analysis in HEM showed a band of 80 kDa that corresponds to the *N*-glycosylated isoform and several smaller bands corresponding to lesser *N*-glycosylated forms of the enzyme [53]. In A375 cells, TYR was also detected but only in its main isoform (Fig. 3a). TYR was absent in ADSC, but after 2 weeks of differentiation, the protein was expressed in PreMel as a result of the induction protocol (Fig. 3a–e). Immunofluorescence of TYR showed a cytoplasmic localization in 12.12% ± 5.2% of the cells (Fig. 3j–m). PMEL also had a cytoplasmic localization as expected, and positive cells were quantified as 10.62% ± 4.3% (Fig. 3n–q). These results showed that the PreMel not only express MITF-A and MITF-M, but also TYR, the main component of the melanin enzymatic machinery, and PMEL, a structural component of melanosomes, to a lesser degree. In summary, 100% of PreMel were MITF^+^ (A or M), around 12% of PreMel were TYR^+^, and 10% of cells were PMEL^+^. Hence, PreMel can be characterized as a mixed population of MITF^+^ cells with subpopulations of TYR^+^ and PMEL^+^ cells.

### Similarities between PreMel and HEM cell cycles

We evaluate the cell cycle of PreMel in vitro as compared with ADSC and HEM. At the end of the exponential phase (day 6), the cell number of ADSC was 2.2× higher than PreMel and HEM, while no difference was detected among those two types. At the end of the growth curve, ADSC reached a cell number 4.3× higher than PreMel and HEM, but the similarities between these two types remained (Additional file 7: Figure S7A). Population doubling time (PDT) and the growth rate (GR) were calculated to demonstrate the similarities between the cell cycles of PreMel and HEM (Additional file 7: Figure S7B).

Likewise, as HEM are exposed daily to physiological injuries, such as UVR and the presence of reactive oxygen species (ROS), we simulated and evaluated the effect of these two parameters over the cell cycle. At 10 or 20 mJ/cm^2^ UVR, both PreMel and HEM were slightly and similarly affected by the radiation without significative changes to either PDT or GR (Additional file 7: Figure S7B). Additionally, we evaluated the effect of oxidative stress over cell proliferation. While all the cells analyzed were affected by the presence of H_2_O_2_, ADSC and PreMel showed similar sensibility to the tested concentrations (Additional file 7: Figure S7C).

### Measurement of TYR activity in PreMel

**Fig. 4:**
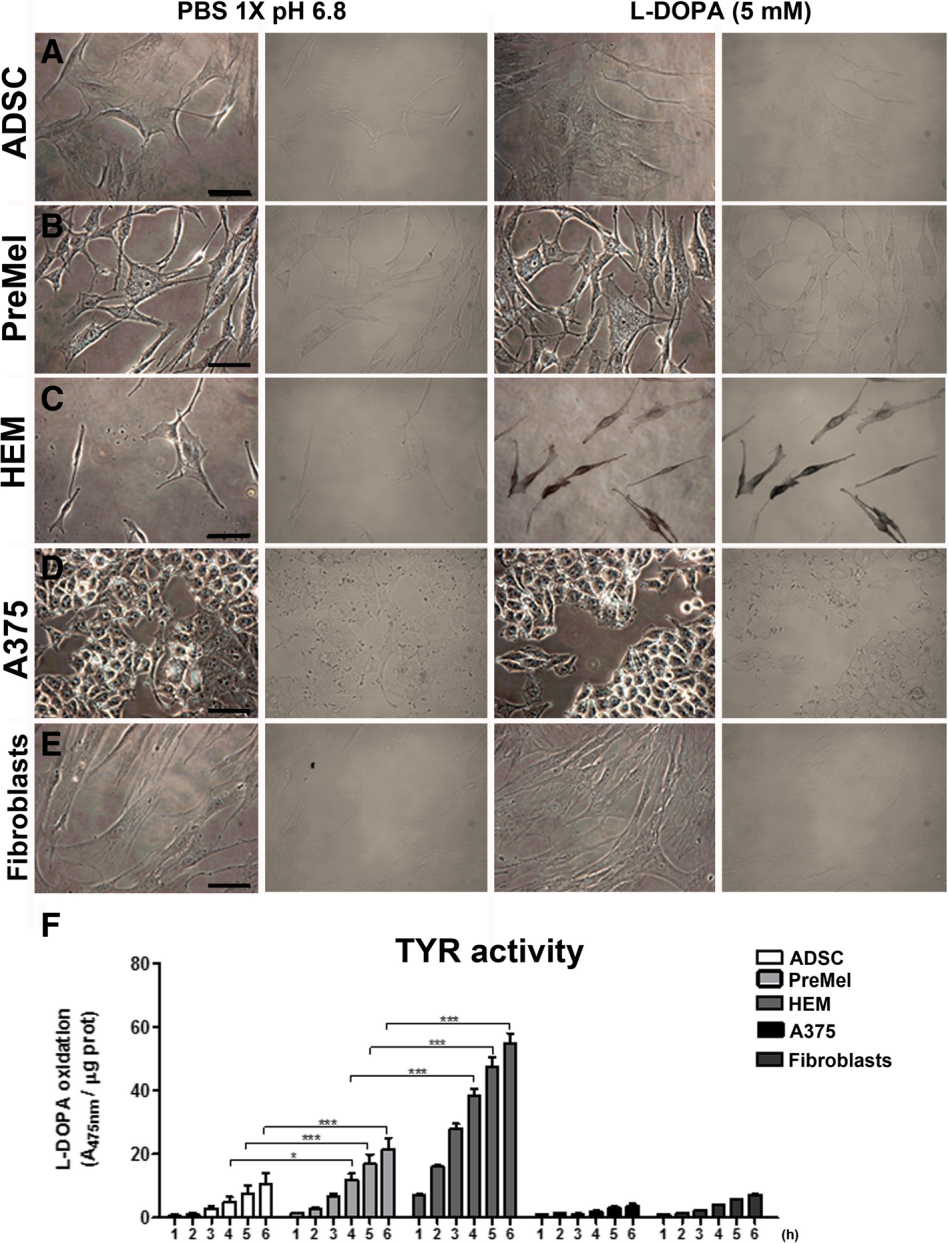
Measurement of TYR activity of PreMel. L-DOPA assay was performed in cell monolayers of **a** ADSC, **b** PreMel, **c** HEM, **d** A375 cells, and **e** fibroblasts. Second and fourth columns show a different microscope filter to enhance the contrast for dark granule visualization. **f** TYR activity was measured by absorbance in the same cell lines at different time points. L-DOPA oxidation was compared in each time point for statistical analysis. *P < 0.05, two-way ANOVA and Bonferroni post-test. ADSC, PreMel, and HEM were compared. Scale bar = 100 μm

TYR is essential for the synthesis of melanin; hence, we evaluated their activity with the L-DOPA assay in PreMel. First, we evaluated the generation of a dark deposit in cell cultures through phase-contrast microscopy. ADSC were negative for dark granules (Fig. 4a), and PreMel showed a slight dark coloration compared with the PBS control (Fig. 4b), but brighter in comparison with the strong dark color in HEM (Fig. 4c). No deposits were distinguished in either A375 cells or fibroblast samples (Fig. 4d, e). As L-DOPA assay visualization by phase-contrast microscopy is unable to detect low levels of TYR activity, we performed a L-DOPA assay in which the oxidation of the substrate was measured by absorbance over time. After 6 h of incubation, there was a significant difference in the absorbance in PreMel showing the oxidative activity of TYR over the background noise that was obtained in ADSC. This result evidences that PreMel express TYR (Fig. 3) in its active form. Between 4 and 6 h post-incubation, a peak in TYR activity was observed in PreMel although it remained below HEM activity in accordance with immunofluorescence data showing that 12% of PreMel were TYR^+^ and that the enzymatic activity detected must come from these specific cells. In fibroblasts and A375 samples, there was a slight increase in absorbance, but this was likely due to the background noise or the L-DOPA oxidation expected over time. No statistical differences were obtained between the two cell types, confirming the assay specificity.

### Evidence of melanin synthesis and melanosome biogenesis

**Fig. 5:**
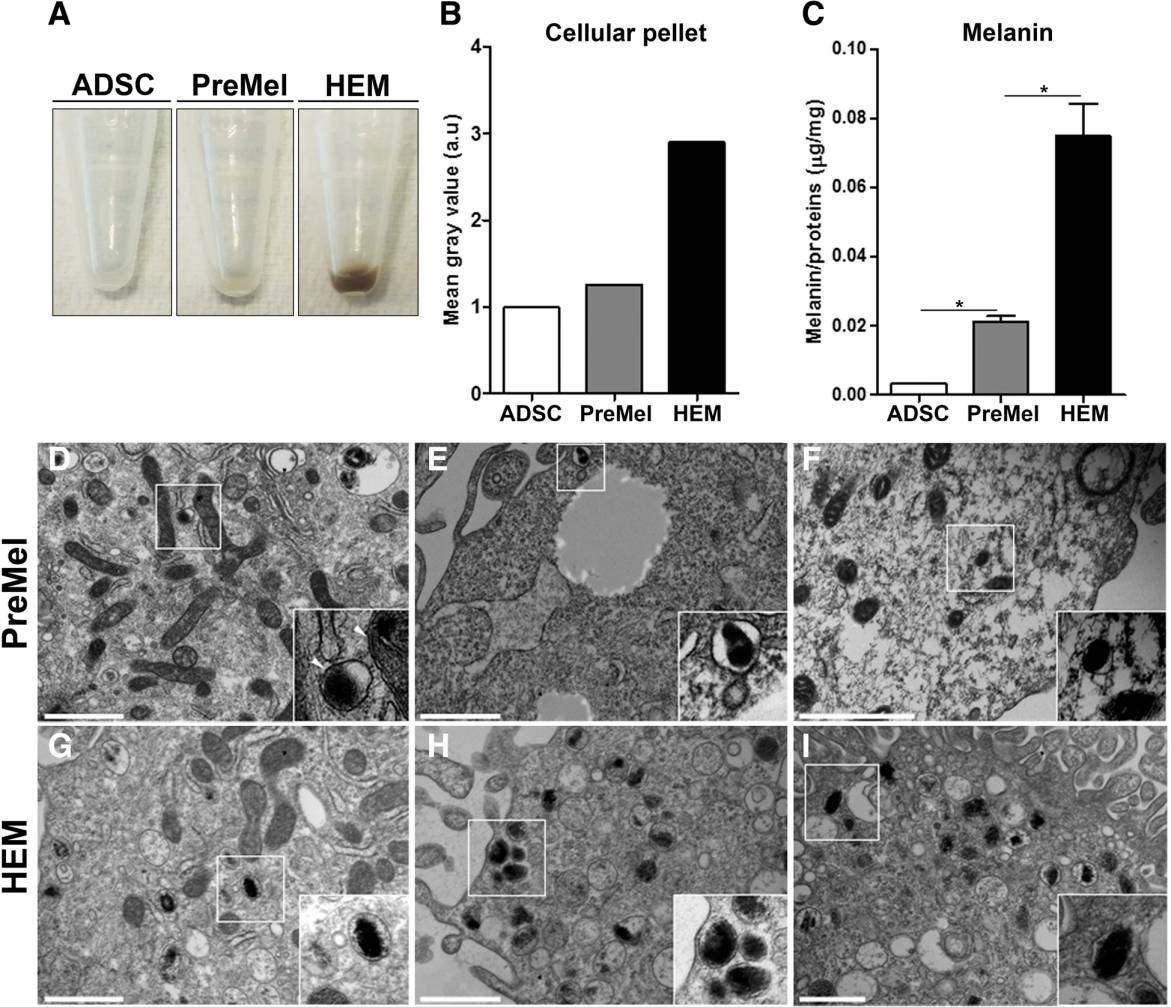
PreMel synthetize melanin and form melanosomes. **a**, **b** Representative cellular pellets of ADSC, PreMel, and HEM and quantification of the respective mean gray value. **c** Melanin content was determined in ADSC, PreMel, and HEM. Pigment is present in PreMel but is significantly lower than in HEM. *P < 0.05 unpaired Student’s *t* test and Welch’s correction. **d**–**f** Ultrastructural analysis shows representative melanosomes in the cytoplasm of PreMel and a higher magnification in the inset. White arrowheads show the single and double membrane of melanosomes and mitochondria, respectively. **g**–**i** Cytoplasmic HEM melanosomes. Scale bar = 250 μm

Melanin is the end product of a series of enzymatic reactions that take place inside the melanosome. Consequently, we evaluated if PreMel were capable of synthetizing melanin in vitro and packaging it into melanosomes. First, we observed that the color of the cellular pellet of PreMel was darker than ADSC (Fig. 5a) and lighter than HEM, and this was correlated with the mean gray value of the pellets (Fig. 5b). Next, we determined the presence of melanin in PreMel lysates using synthetic melanin as a standard, and we showed an absorbance significantly higher than ADSC (unspecific absorbance at the respective wavelength) but lower than HEM. This again confirmed that PreMel are functionally closer to HEM and possess the capacity to synthesize melanin. (Fig. 5c). Remarkably, two cytokines related with whitening effects of ADSC in the skin through TYR inhibition activity, *TGF-β1* and *IL-6*, were downregulated after the 4-week differentiation protocol in PreMel (Additional file 8: Figure S8).

Conversely, we analyzed the ultrastructure of PreMel by transmission electronic microscopy (TEM) and identified melanosomes as single-membrane vesicles with dark pigment inside (Fig. 5d–f), similar to the structures present within the HEM cytoplasm (Fig. 5g–i). Such structures were not observed in ADSC (data not shown). We were able to detect melanosomes in PreMel; however, the number of melanosomes in PreMel was considerably lower than in HEM. In HEM, the four stages of melanosome biogenesis (I–IV) were identified, but in PreMel, only stages III and IV were distinguished. Earlier stages could be mistakenly confused with other intracellular formations such as secretory vesicles. Both results support the hypothesis that PreMel represent a population of melanocyte-like cells with melanin synthesis capabilities in vitro.

### Engraftment of PreMel in mouse skin and synthesis of melanin in vivo

**Fig. 6:**
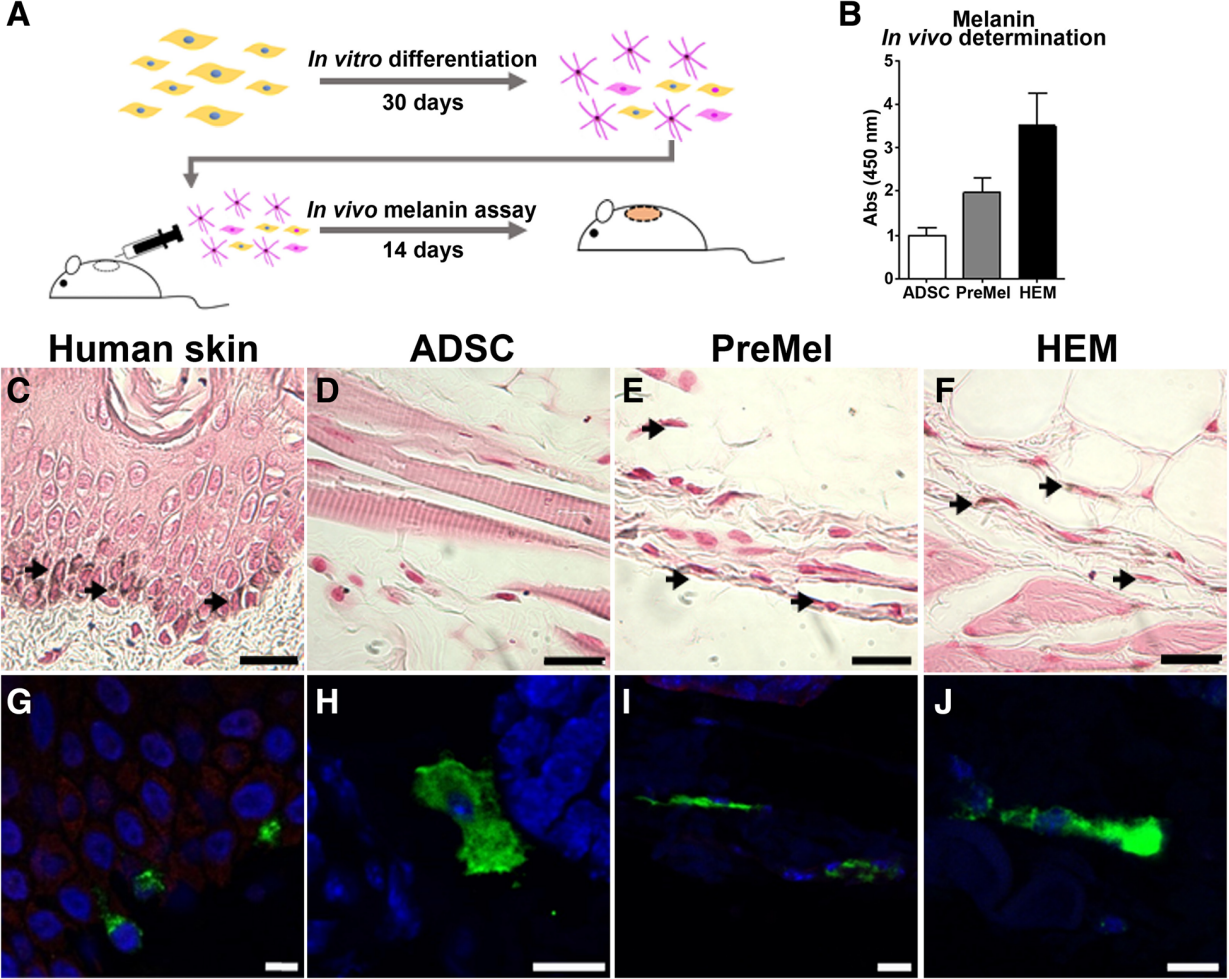
Transplanted PreMel produce melanin and express TYR in vivo. **a** Schematic representation of PreMel differentiation and the in vivo setup. 1 × 10^6^ cells (ADSC, PreMel, or HEM) were injected intradermally into the back skin of NSG mice, and melanin production was evaluated after 14 days. **b** Melanin determination in NSG mouse skin shows that PreMel synthetize melanin in vivo to a lesser extent than HEM. Fontana-Masson staining for melanin in the human skin as a positive control (**c**). Black arrows show HEM in situ in the basal membrane of the epidermis. **d** Mice treated with ADSC are negative for melanin staining. **e** PreMel are positive for the Fontana-Masson staining (indicated by black arrows). **f** HEM injected in the mouse skin are positive for melanin, (scale bar = 100 μm). The presence of injected cells was also determined by TYR immunofluorescence in the human skin (**g**) and in mouse skin slides. **h** TYR-negative ADSC were stained with anti-human CD90 to demonstrate their presence. **i** Similar to in vitro samples, PreMel are positive for TYR in the mouse skin, demonstrating that the melanin synthesis machinery is active in differentiated cells in vivo. **j** Injected HEM are also positive for TYR in this in vivo setting (scale bar = 10 μm)

PreMel were injected in the back skin of immunodeficient mice in order to visualize their HEM-like properties in vivo (Fig. 6a), for this experimental setup, we used albino NSG animals because they lack functional epidermal melanocytes [54]. After 2 weeks, the skin was recovered for histological analysis. First, we quantify the presence of melanin in the skin, and we were able to determine that in fact, PreMel synthetize melanin in vivo, but to a lesser extent with respect to HEM (Fig. 6b), possibly because a minor proportion of PreMel has the potential of synthesize and package the melanin (MITF^+^ TYR^+^ PMEL^+^ cells). In order to discard any unspecific measurement, we analyzed microscopically the presence of PreMel. For this, skin slides were stained with the Fontana-Masson stain using human skin as a positive control, where HEM in the basal membrane of the dermis were stained brownish-dark (Fig. 6c). While no stained cells were detected in the animals injected with ADSC (Fig. 6d), we were able to distinguish positive stained cells in the PreMel-treated animals which demonstrated that injected cells were in fact cells with HEM-like properties (Fig. 6e), reflecting that PreMel behave as HEM in vivo (Fig. 6f). Next, we determined the expression of TYR in PreMel in vivo in the mouse skin, using human skin (Fig. 6g) and injected HEM (Fig. 6f) as positive controls. Notably, we detected TYR^+^ cells in the skin of the animal, cells that were identified as injected PreMel (Fig. 6h), because TYR was not detected in NSG skin control (data not shown). In animals injected with ADSC, TYR was not detected; however, CD90 was used in order to confirm the presence of the injected human cells (Fig. 6h).

## Discussion

To date, the current approach for cell-based therapy for vitiligo is to obtain a large number of HEM in order to replace the lost cells. Although this invasive strategy has shown relative success, multiple biopsies and repetitive treatments are required to obtain the necessary number of cells to complete the entire treatment [11, 12]. Hence, the generation of HEM or their precursors that can reach function completely in situ is an attractive strategy worth exploring.

Mimicking the conditions of the HEM microenvironment at the molecular level is key for a successful differentiation in vitro. We developed an inductive medium which includes ligands for some of the fundamental pathways involved in HEM differentiation. FGF2 is secreted by keratinocytes [55] and is a potent mitogen for HEM in conjunction with DBcAMP [56], as well as a fundamental role player in HEM differentiation through the activation of *PAX3* transcription through ERK1-dependent STAT3 phosphorylation [55]. DBcAMP penetrates the plasma membrane more easily than cAMP [57] and acts as a potent mitogen and can induce melanoblasts differentiation into HEM in vitro [56, 58]. αMSH, also secreted by keratinocytes, signals through MC1R and induces an increase in the cAMP levels which activates PKA. Through PKA activation, several MC1R-dependent functions are translated in HEM, including melanin synthesis. Hydrocortisone promotes cell differentiation in HEM cultures [59, 60], and dexamethasone, a synthetic form of it, has been used for ESC differentiation into HEM along with FGF2 and an enhancer of cAMP levels such as DBcAMP [21]. Insulin, transferrin, and selenium have been reported as the supplemental factors for low-serum media [30] but has been shown that insulin in combination with FGF2 promotes HEM proliferation [61]. The final formulation for our inductive medium was able to effectively recapitulate the HEM developmental stages at a shorter time scale, and consequently, rapid differentiation of ADSC into PreMel was achieved within only 30 days. Ligands from other signaling pathways, such as WNT and endothelin [62, 63] can be added to this protocol in order to increase the proportion of terminally differentiated HEM or to use the co-culture with keratinocytes and/or fibroblasts, which could represent the in vivo niche in an in vitro setting promoting the development and differentiation of PreMel.

The mechanism of differentiation of PreMel in response to the inductive media was explained through the analysis of specific genes expressed in HEM. *PAX3*, a member of the PAX family, is the first transcription factor that was upregulated in PreMel and is also one of the described factors responsible for the commitment of melanoblasts from a neural crest cell, along with others including SOX9, SOX10, FOXD3, SNAI2, and MITF [64, 65]. PAX3 recognizes specific domains of the MITF promotor [66] and is in part responsible for MITF transcription. An increase in *PAX3* expression in PreMel was accompanied with an increase in *MITF* levels starting during the second week of induction. This could also be a result of the presence of other transcription factors described for this role in HEM, such as SOX10, LEF1 (activated by WNT/β-catenin signaling), and CREB, not analyzed in this work. Remarkably, *MITF-A* levels were higher than *MITF-M* levels, which could be explained for the differential accessibility to the alternative promoters that are present in the MITF gene, being the most proximal to the transcriptional start site (TSS) the one for MITF-M, the shortest isoform of the gene [51, 67]. This result suggests that the increase in MITF protein levels is mostly represented by the A isoform and that the expression of HEM-specific genes are commanded by MITF-M, despite its lower expression levels.

An increase in *MLANA* and *TRP2* during the third week was observed along with the increases in *PAX3* and *MITF-M*, which suggests that the transcriptional activity of the induced levels of MITF-M in PreMel are responsible for *MLANA* and *TRP2* expression, as it has been described that PAX3 cooperates with MITF-M in transcription of TRP2 [68].

On the other hand, MC1R, the main regulator of melanin synthesis in HEM [69], was expressed at low levels in ADSC but was upregulated starting in the second week. It is possible that MC1R activation is independent of MITF and is instead the outcome of a positive feedback loop linked to the presence of DBcAMP and αMSH in the induction culture media [70]. *MC1R* displayed a two- to threefold increase in the transcript levels in PreMel, compared to ADSC. The expression level remained low (from 0.18 to 0.44) in agreement with the relatively low number of MC1R molecules expressed at the HEM surface [71]. Notably, MC1R is a GPCR (G protein-coupled receptor) and can undergo desensitization and internalization via the endocytic pathway [70], meaning that the continued presence of αMSH during the differentiation protocol does not ensure continuous signaling by MC1R.

In addition to the molecular data, the expression of MITF, TYR, and PMEL allow us to estimate a differentiation yield in PreMel: 100% of PreMel were MITF^+^ (A or M), around 12% of PreMel were TYR^+^, and 10% of cells were PMEL^+^. This suggests that the entire PreMel population could be committed to the HEM lineage (MITF^+^ TYR^−^ PMEL^−^), while only a subpopulation is terminally differentiated as HEM (MITF^+^ TYR^+^ PMEL^+^) and possess melanin synthesis capabilities.

Another relevant parameter evaluated to evidence cell differentiation was the expression of classic MSC markers. According to Dominici et al. [46], CD105 is listed within the stem cell-associated markers necessary to appropriately identify MSC. While ADSC showed high CD105 expression, following the differentiation induction protocol, the expression diminished almost completely in PreMel. CD105, or endoglin, is a type I transmembrane glycoprotein and is part of the TGF-β receptor complex which controls several cellular functions in HEM, including melanin synthesis. TGF-β1 inhibits melanin formation through multiple mechanisms [72]: in immortalized melanocytes (Mel-Ab cells), TGF-β1 treatment diminishes TYR activity, and consequently, melanin synthesis decreases; TGF-β1 signaling not only reduces the MITF activity promotor, but also decreases the levels of MITF, TYR, TRP1, and TRP2 proteins [73, 74]. Therefore, diminished CD105 expression in PreMel after the induction protocol could be required for the commitment of PreMel to the epidermal lineage. In fact, *TGF-β1* transcript levels also decrease after 4 weeks along with *IL-6*, another cytokine described as an inhibitor of melanin synthesis [75]. Additionally, CD73 (ecto-5′-nucleotidase) is another classic positive marker of ADSC, and there is no clear correlation between its diminished expression in PreMel and HEM, because there are few reports of CD73 in normal HEM. CD73 is a target of TGF-β signaling in immune cells (murine CD4^+^ T and CD8^+^ T cells, dendritic cells, and macrophages) [76] and the reduced signaling of this pathway in PreMel due to the lower *TGF-β1* expression, and CD105 levels could be responsible for the decrease in CD73 expression.

The similarities in the expression of CD105 and CD73 between PreMel and HEM, and the functional consequences that may result in diminished signaling by the TGF-β pathway for the absence of CD105, lead us to propose that CD105^neg^ CD73^low^ cells represent a viable alternative to replace the lost HEM in vitiligo. We identify CD105^neg^ CD73^low^ as a heterogeneous cell population expressing MITF, TYR, and PMEL composed of (1) ADSC deriving from the MSC lineage but with functional properties allowing for differentiation and immunomodulation and (2) PreMel with a precursor-like phenotype and the potential to mature into HEM, both of which located at the niche of the epidermis could play a dual role in the treatment of vitiligo: ADSC playing a regulator function against the inflammatory environment of the pathological skin, and PreMel providing a source of melanin-producing cells that could reach a terminal differentiation in response to components of the microenvironment.

Indeed, ADSC have been used in vitiligo as a co-adjuvant of HEM in vitro and in humans in an autologous cell transplant where it has been reported that a combined injection of both augments the proper settlement of HEM in the grafted area [77]. Interestingly, the use of follicular-derived MSC improves survival of HEM by reducing the inflammation within the treated area [12]. Additionally, it has already been reported that dermal mesenchymal stem cells (DMSC) inhibit the homing of lymphocytes CD8^+^ to the perilesional area in the human skin [78]. Hence, our future investigation will focus on deciphering the effect of partially differentiated ADSC on immunosuppression in the inflammatory environment of patients’ skin.

Besides the differentiation yield, temporality is relevant when clinical settings are considered. Our differentiation protocol is 150 days shorter than previously published HEM differentiation methods from MSC [40, 42] and is capable of generating a sufficient number of PreMel cells for possible clinical settings. Starting with a 50-mL sample of lipoaspirate, which requires just one ambulatory procedure, we obtained 9 × 10^6^ cells in primary culture with a differentiation protocol started after four to five subcultures of continuous cell proliferation. Additionally, during the differentiation process, the proliferation continues, resulting in 150 times the initial number of cells. Taking into consideration the accessibility of the autologous cell source and the required short ex vivo procedure, fat tissues could be easily harvested from vitiligo patients for ADSC isolation, differentiation, and transplantation in the depigmented skin areas.

## Conclusions

We show in this work a short and scalable protocol for obtaining HEM replacement cells as an alternative for the treatment of vitiligo. An accessible and minimally invasive source for adult stem cells was used allowing the possibility for autologous treatments. However, additional effort is required to drive these cells along the translational pathway. This includes the assessment of possible undesired modification of the cells due to ex vivo manipulation following the established in vitro process. Nevertheless, we believe that PreMel open a new therapeutic window for a disease that has been deferentially referred to as “just a cosmetic issue,” and give new hope for vitiligo patients.

## Additional files

Additional file 1: **Figure S1.** ADSC characterization. Expression of classical MSC markers was evaluated by flow cytometry. ADSC are positive for (A) CD105, (B) CD90, (C) CD73, (D) CD44, and (E) CD13. Additionally, ADSC are negative for (F) CD31, (G) CD14, (H) CD45, and (J) CD106. I) CD34 was partially positive as has been described for ADSC. Autofluorescence controls are shown in blue. ADSC have the potential to differentiate into (K) chondrogenic lineage, stained with Safranin O; (L) osteogenic lineage, with Alizarin Red S positive staining; and (M) adipogenic lineage, with cells positive for Oil Red O stain. (Scale bar = 100 μm). N) Clonogenic potential of ADSC measured by CFU-F. Colonies composed of more than 50 cells, in 5 dilutions, were quantified. Absorbance of total CV staining was also graphed, *n* = 3. (JPG 2140 kb)

Additional file 2: **Figure S2.** Gating strategy for flow cytometry analysis. We show representative plots for CD105 expression after the gating of ADSC, PreMel and HEM in the SSC-A vs FSC-A graph, as well as the posterior elimination of doublets. (JPG 820 kb)

Additional file 3: **Figure S3.** Expression of MSC-related markers. A) Dot plot shows CD44 expression in ADSC, PreMel and HEM. B) MFI ratio for CD44. C) Expression of CD90. D) MFI ratio for CD90. E) Expression of CD146. F) MFI ratio for CD146. G) Expression of CD117. H) MFI ratio for CD117. MFI ratio was calculated as MFI (specific staining)/ MFI (autofluorescence) for each marker. Data from PreMel and HEM were normalized with respect to undifferentiated ADSC. For dot plots, gray dots represent autofluorescence control and black dots represent stained cells with their respective marker n = 3, *P < 0.05 One-way ANOVA. (JPG 168 kb)

Additional file 4: ** Figure S4.** Reduction of the adipogenic differentiation potential in PreMel. ADSC and PreMel were stimulated for adipogenic differentiation. Red Oil O staining in A) ADSC and B) PreMel. C) Oil Red O^+^ cells quantification shows that the adipose potential is diminished in PreMel respect to ADSC. Scale bar = 500 μm. *P < 0.05 unpaired Student’s *t* test. (JPG 941 kb)

Additional file 5: **Fig. S5.** Gene expression of HEM-related genes. *SOX2*, *SOX10*, *PAX3*, *MITF-A*, *MITF-M*, *MC1R*, *PMEL*, *TRP2*, *TYR*, and *MLANA* were evaluated in A) HEM and B) A375 cells, an amelanotic melanoma cell line. (JPG 399 kb)

Additional file 6: **Figure S6.** MITF expression in PreMel and HEM. A) Representative image of MITF immunofluorescence in PreMel shows that the entire population expresses the protein. B) The same result was obtained through cell cytometry analysis. MITF expression in HEM by C) immunofluorescence and D) cell cytometry. Scale bar = 100 μm. (JPG 453 kb)

Additional file 7: **Figure S7.** PreMel show similar cell proliferation and stress resistance compared to HEM. A) Growth curve of ADSC, PreMel and HEM shows that the PreMel growth pattern is similar to that of HEM. PDT (population doubling time) and GR (growth rate) were estimated from the data in (A). Cells were exposed to UVR and PDT was estimated during the exponential growth phase. B) A lower PDT in PreMel than in HEM indicates that ADSC are more resistant to UVB radiation. The PDTs of PreMel and HEM are not different after irradiation with 10 or 20 mJ/cm^2^. ADSC have the highest growth rate, whereas PreMel’ is lower and similar to that of HEM (n = 3, *P < 0.05 One-way ANOVA. C) ADSC are sensitive to ROS in all concentrations. At low concentrations of H_2_O_2_, PreMel and ADSC show similar resistance (n = 3, *P < 0.05 two-way ANOVA). (JPG 720 kb)

Additional file 8: **Figure S8.** Levels of whitening-related cytokines. *IL-6* and *TGF-β1* levels were evaluated after the 4-week differentiation protocol; they are diminished compared to undifferentiated ADSC. (JPG 263 kb)

## Data Availability

The datasets analyzed during the current study are available from the corresponding author on reasonable request.

## Abbreviations

ADSC: Adipose-derived stem cells
HEM: Human epidermal melanocytes
MITF: Microphthalmia-associated basic helix-loop-helix leucine zipper transcription factor
MSC: Mesenchymal stem cells
PreMel: Pre-melanocytes
TYR: Tyrosinase
