# Supplementary figures and images for: Differentiation of adipose-derived stem cells to functional CD105neg CD73low melanocyte precursors guided by defined culture condition
Source: Stem Cell Res Ther. 2019 Aug 9;10:249. doi: 10.1186/s13287-019-1364-0 (PMC6688240; doi:10.1186/s13287-019-1364-0)

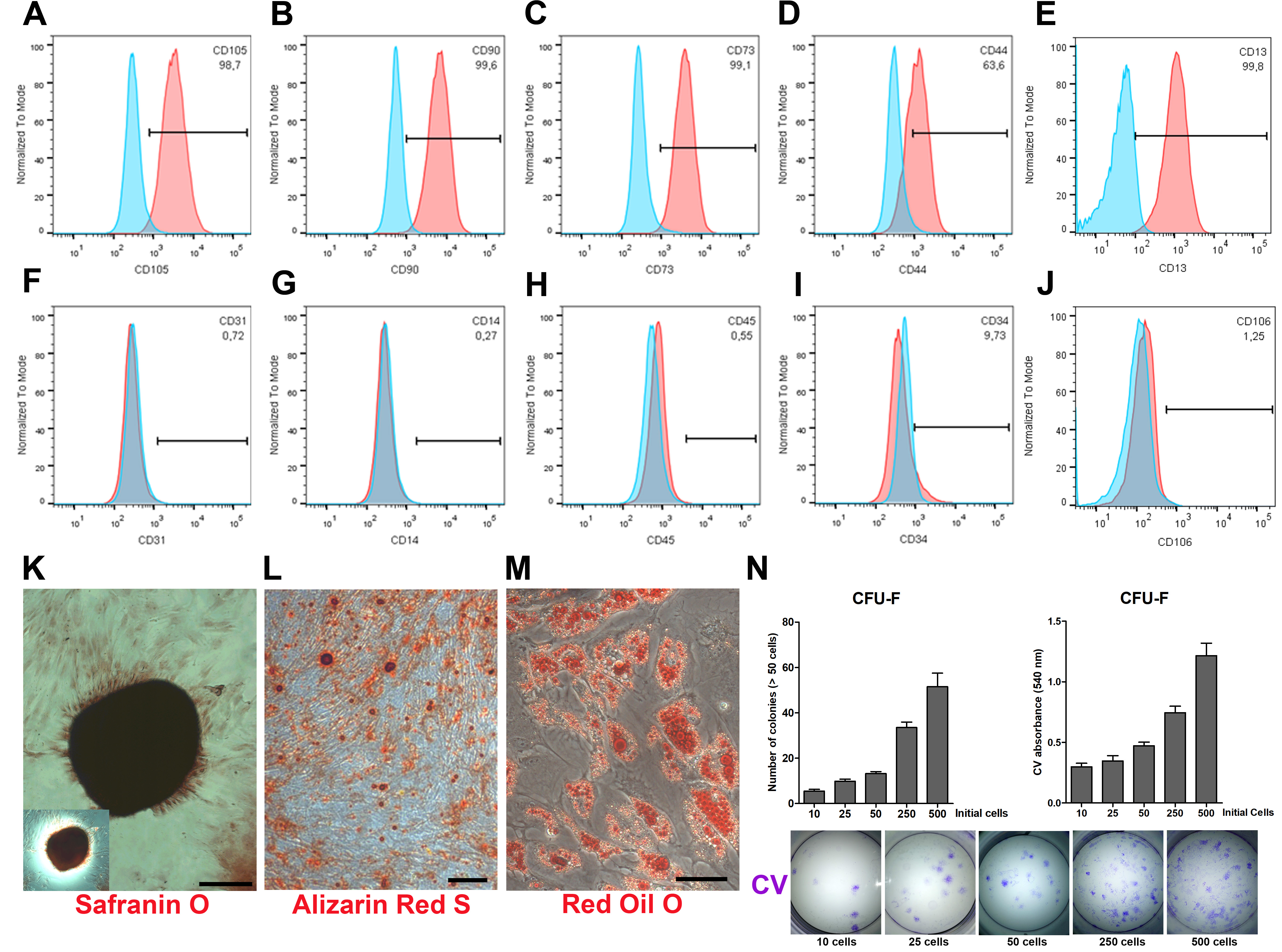

Supplement: Supplementary file 1 — Figure S1. ADSC characterization. Expression of classical MSC markers was evaluated by flow cytometry. ADSC are positive for (A) CD105, (B) CD90, (C) CD73, (D) CD44, and (E) CD13. Additionally, ADSC are negative for (F) CD31, (G) CD14, (H) CD45, and (J) CD106. I) CD34 was partially positive as has been described for ADSC. Autofluorescence controls are shown in blue. ADSC have the potential to differentiate into (K) chondrogenic lineage, stained with Safranin O; (L) osteogenic lineage, with Alizarin Red S positive staining; and (M) adipogenic lineage, with cells positive for Oil Red O stain. (Scale bar = 100 μm). N) Clonogenic potential of ADSC measured by CFU-F. Colonies composed of more than 50 cells, in 5 dilutions, were quantified. Absorbance of total CV staining was also graphed, n = 3. (JPG 2140 kb) [file 13287_2019_1364_MOESM1_ESM.jpg]

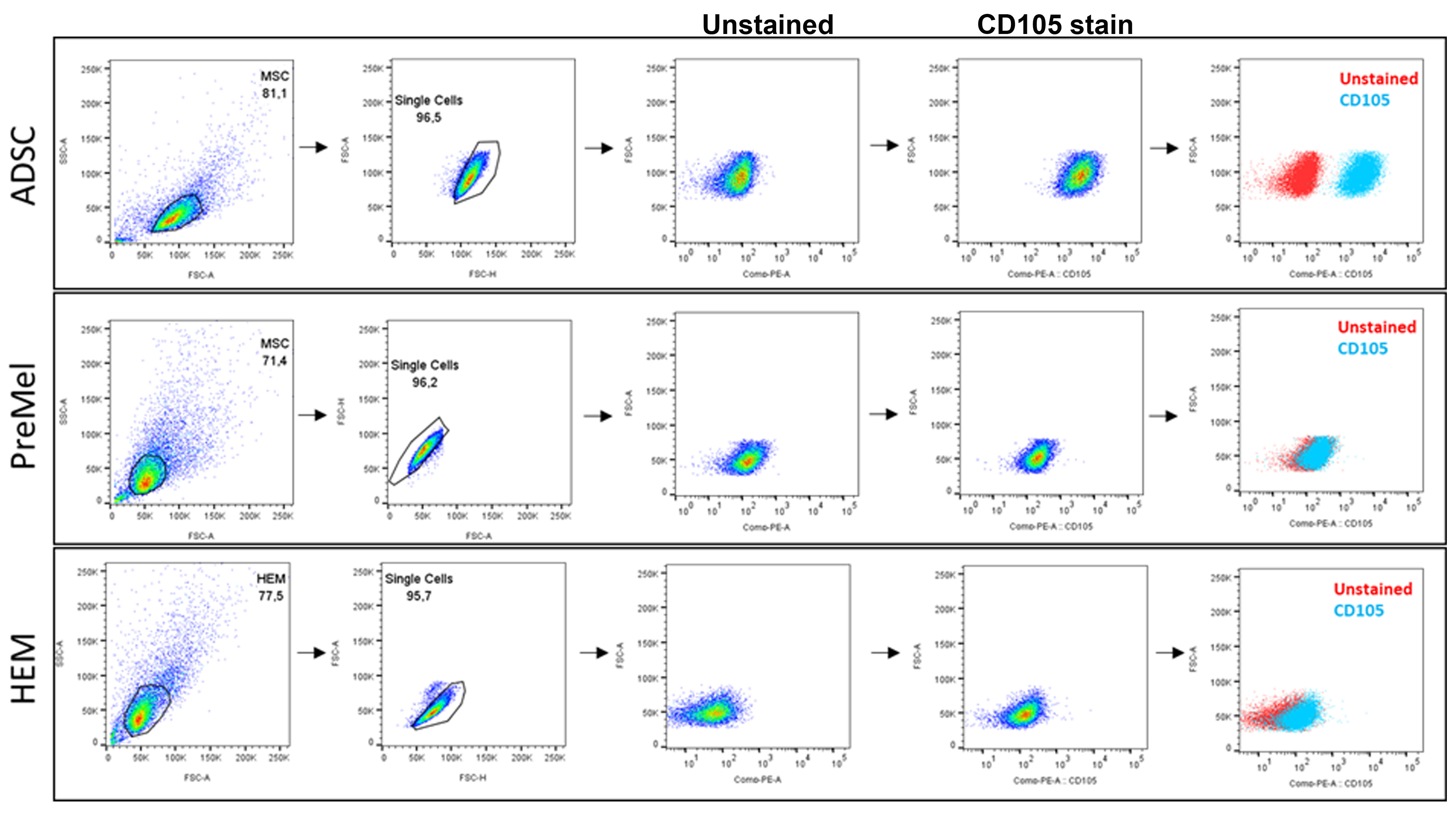

Supplement: Supplementary file 2 — Figure S2. Gating strategy for flow cytometry analysis. We show representative plots for CD105 expression after the gating of ADSC, PreMel and HEM in the SSC-A vs FSC-A graph, as well as the posterior elimination of doublets. (JPG 820 kb) [file 13287_2019_1364_MOESM2_ESM.jpg]

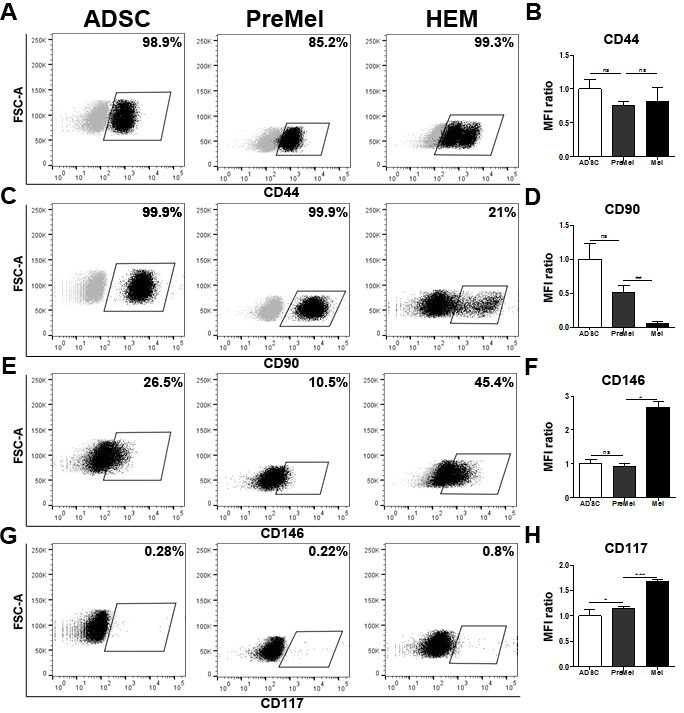

Supplement: Supplementary file 3 — Figure S3. Expression of MSC-related markers. A) Dot plot shows CD44 expression in ADSC, PreMel and HEM. B) MFI ratio for CD44. C) Expression of CD90. D) MFI ratio for CD90. E) Expression of CD146. F) MFI ratio for CD146. G) Expression of CD117. H) MFI ratio for CD117. MFI ratio was calculated as MFI (specific staining)/ MFI (autofluorescence) for each marker. Data from PreMel and HEM were normalized with respect to undifferentiated ADSC. For dot plots, gray dots represent autofluorescence control and black dots represent stained cells with their respective marker n = 3, *P < 0.05 One-way ANOVA. (JPG 168 kb) [file 13287_2019_1364_MOESM3_ESM.jpg]

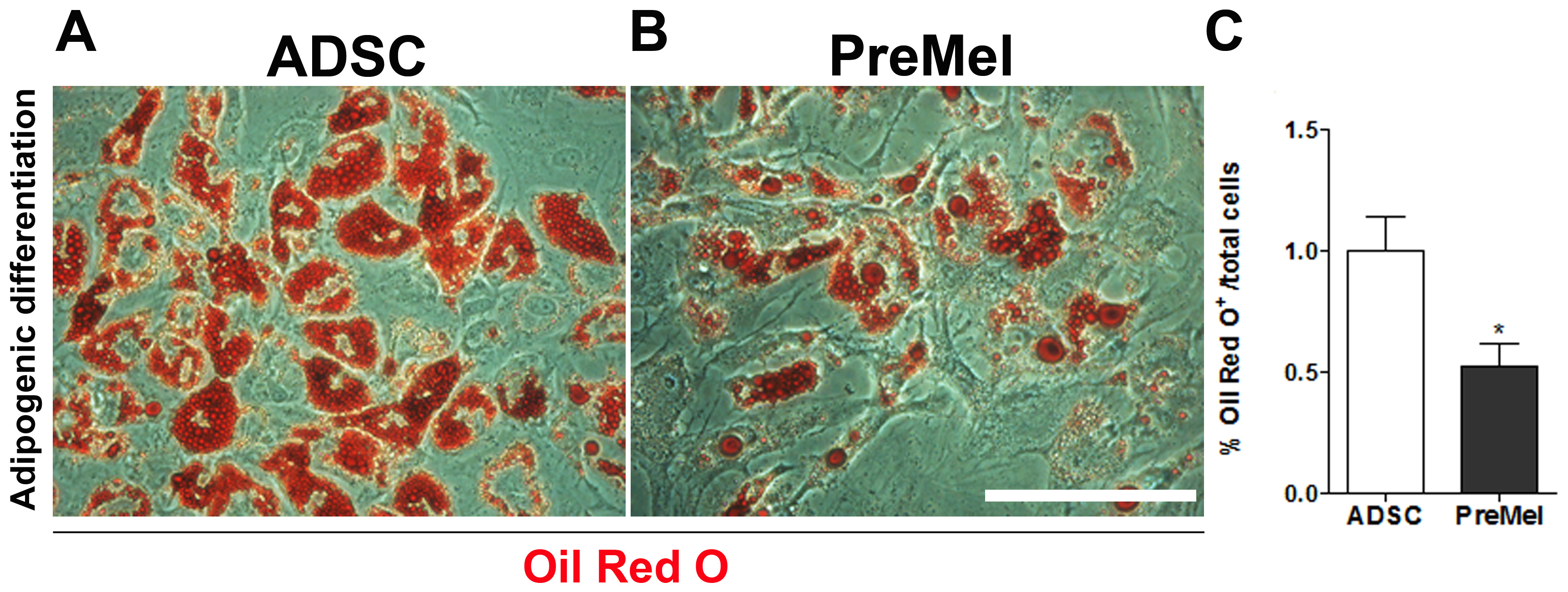

Supplement: Supplementary file 4 — Figure S4. Reduction of the adipogenic differentiation potential in PreMel. ADSC and PreMel were stimulated for adipogenic differentiation. Red Oil O staining in A) ADSC and B) PreMel. C) Oil Red O+ cells quantification shows that the adipose potential is diminished in PreMel respect to ADSC. Scale bar = 500 μm. *P < 0.05 unpaired Student’s t test. (JPG 941 kb) [file 13287_2019_1364_MOESM4_ESM.jpg]

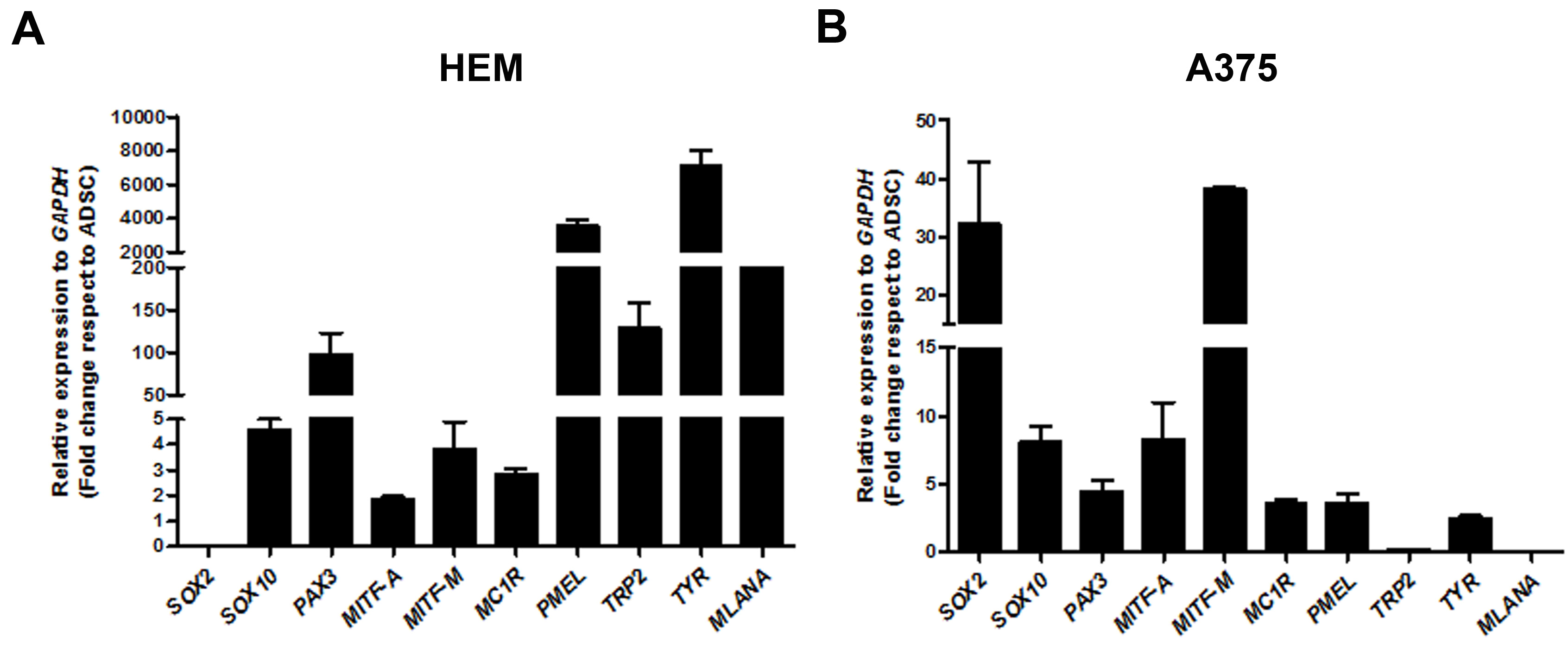

Supplement: Supplementary file 5 — Fig. S5. Gene expression of HEM-related genes. SOX2, SOX10, PAX3, MITF-A, MITF-M, MC1R, PMEL, TRP2, TYR, and MLANA were evaluated in A) HEM and B) A375 cells, an amelanotic melanoma cell line. (JPG 399 kb) [file 13287_2019_1364_MOESM5_ESM.jpg]

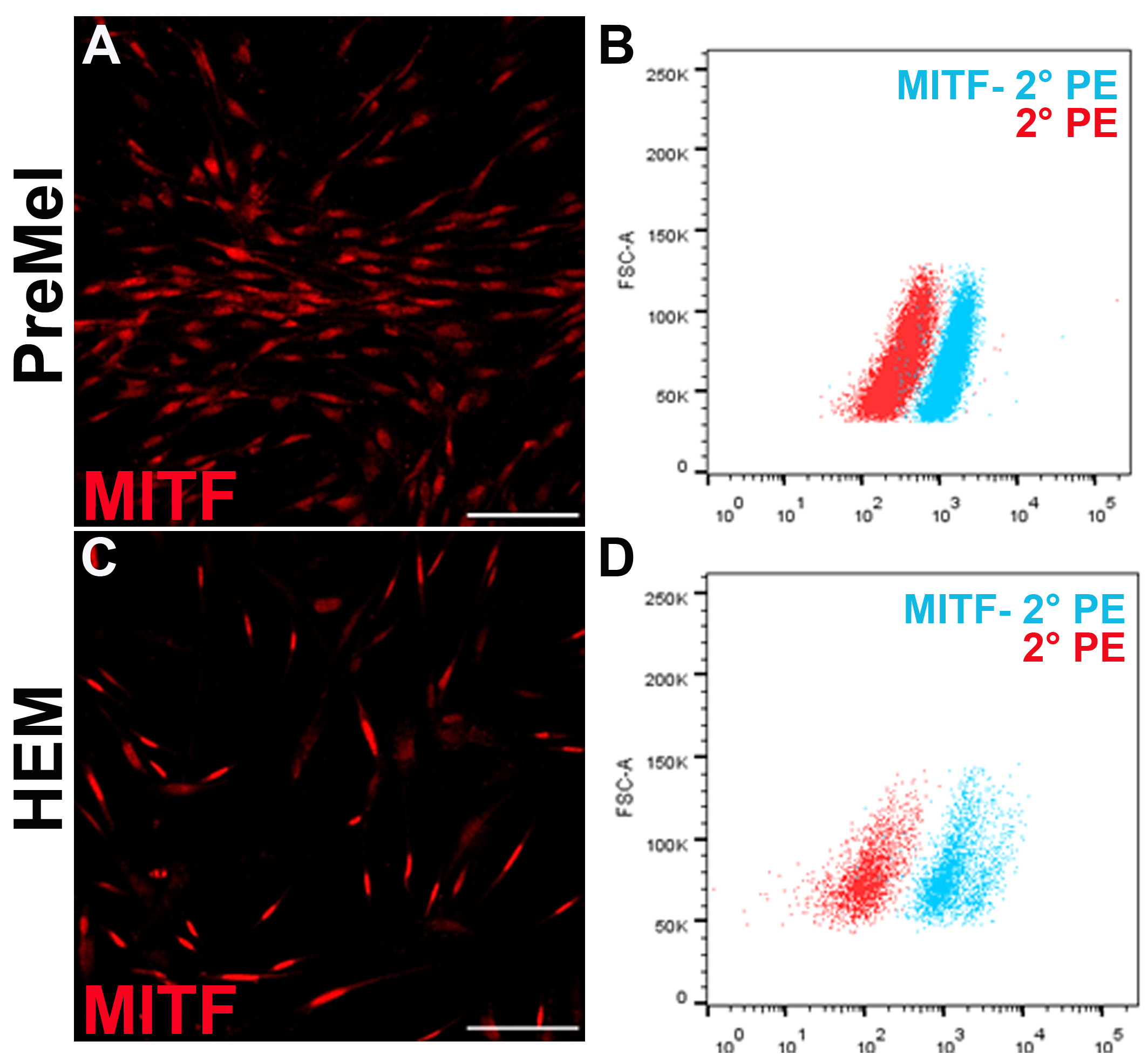

Supplement: Supplementary file 6 — Figure S6. MITF expression in PreMel and HEM. A) Representative image of MITF immunofluorescence in PreMel shows that the entire population expresses the protein. B) The same result was obtained through cell cytometry analysis. MITF expression in HEM by C) immunofluorescence and D) cell cytometry. Scale bar = 100 μm. (JPG 453 kb) [file 13287_2019_1364_MOESM6_ESM.jpg]

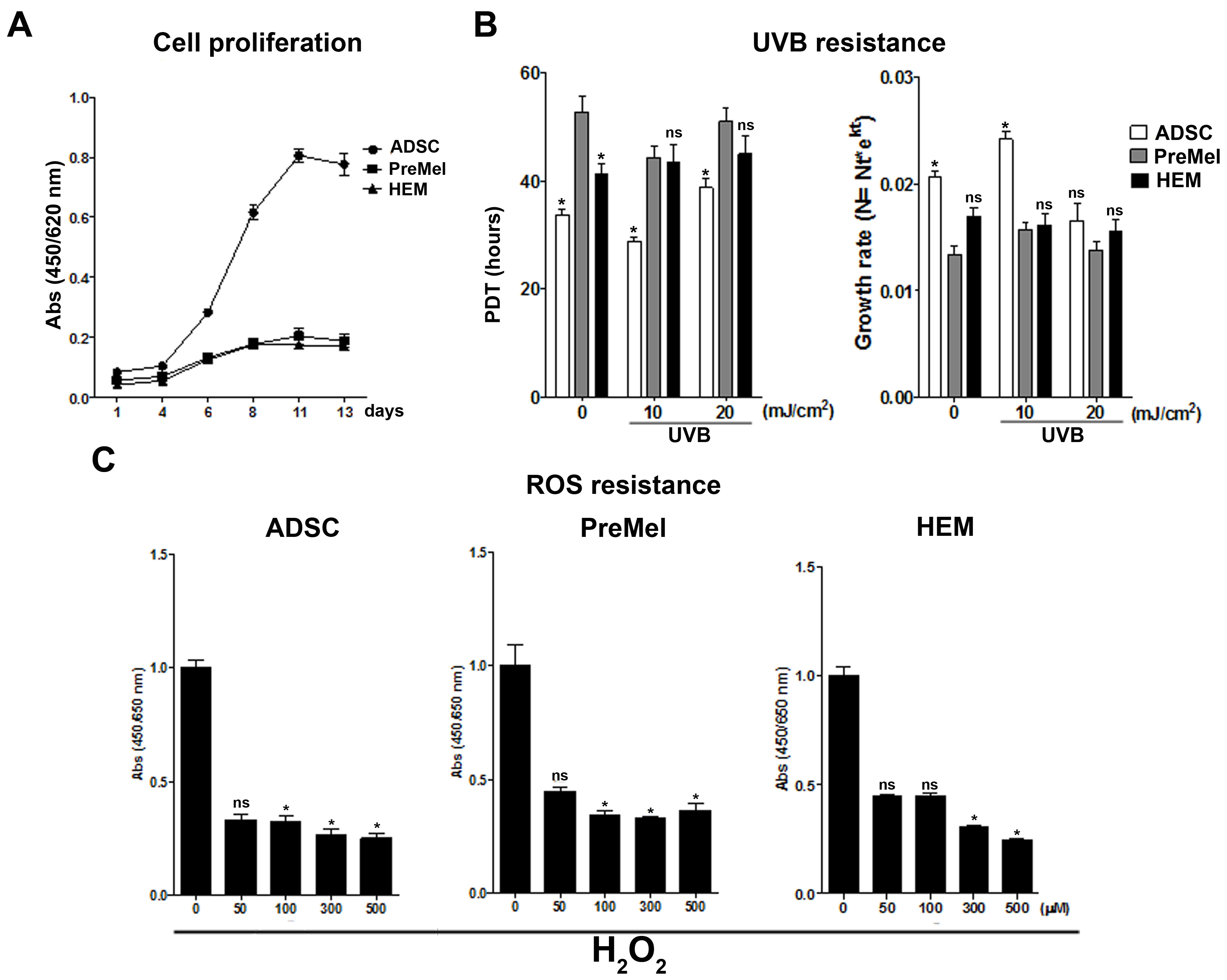

Supplement: Supplementary file 7 — Figure S7. PreMel show similar cell proliferation and stress resistance compared to HEM. A) Growth curve of ADSC, PreMel and HEM shows that the PreMel growth pattern is similar to that of HEM. PDT (population doubling time) and GR (growth rate) were estimated from the data in (A). Cells were exposed to UVR and PDT was estimated during the exponential growth phase. B) A lower PDT in PreMel than in HEM indicates that ADSC are more resistant to UVB radiation. The PDTs of PreMel and HEM are not different after irradiation with 10 or 20 mJ/cm2. ADSC have the highest growth rate, whereas PreMel’ is lower and similar to that of HEM (n = 3, *P < 0.05 One-way ANOVA. C) ADSC are sensitive to ROS in all concentrations. At low concentrations of H2O2, PreMel and ADSC show similar resistance (n = 3, *P < 0.05 two-way ANOVA). (JPG 720 kb) [file 13287_2019_1364_MOESM7_ESM.jpg]

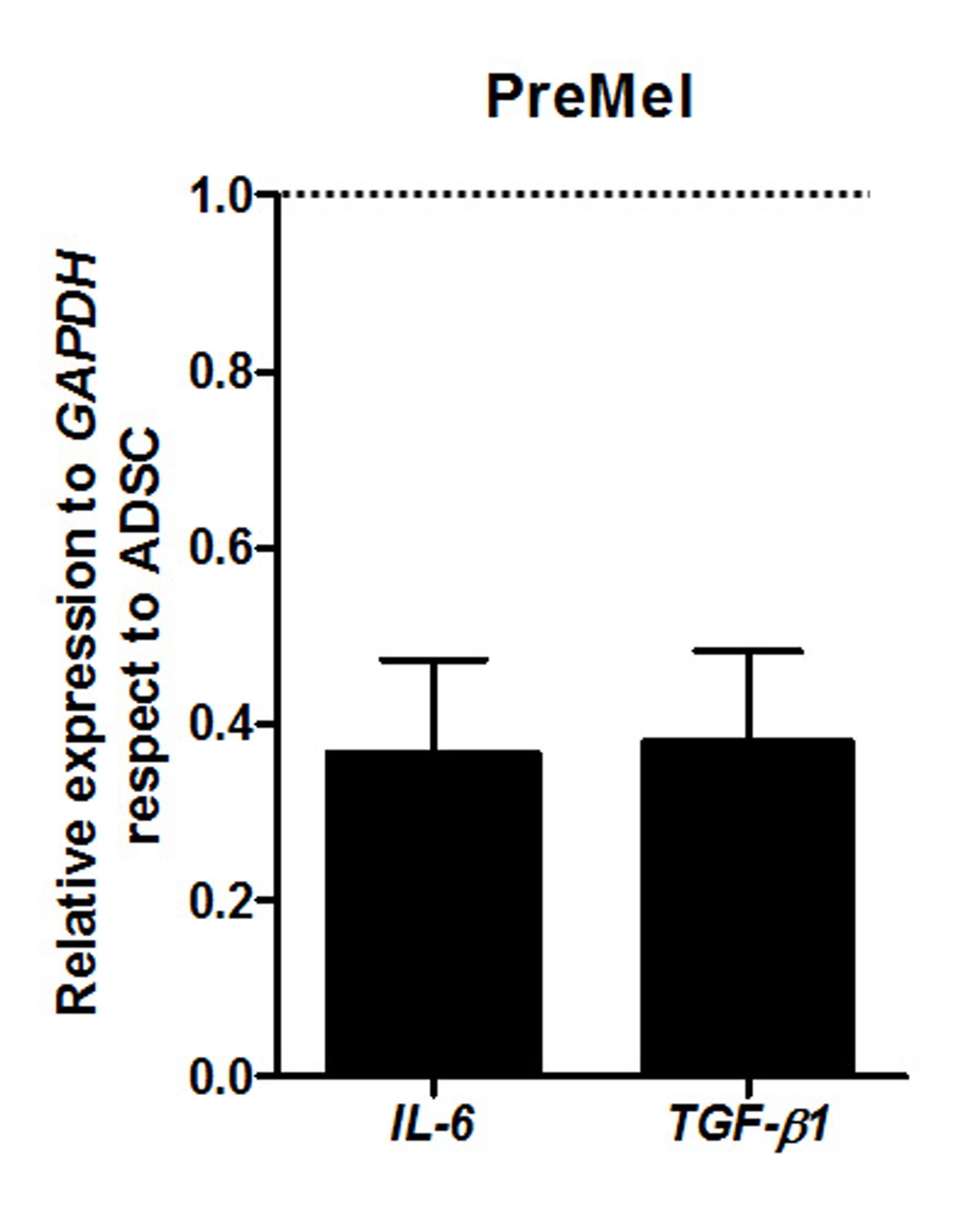

Supplement: Supplementary file 8 — Figure S8. Levels of whitening-related cytokines. IL-6 and TGF-β1 levels were evaluated after the 4-week differentiation protocol; they are diminished compared to undifferentiated ADSC. (JPG 263 kb) [file 13287_2019_1364_MOESM8_ESM.jpg]
